# Changes in the transformative potential of action proposals in Finnish Red Lists from 1986 to 2019

**DOI:** 10.1111/cobi.70312

**Published:** 2026-05-06

**Authors:** Anni Arponen, Anna Salomaa, Henna Fabritius, Aino Juslén, Saija Kuusela

**Affiliations:** ^1^ Research Group Politics of Nature and the Environment (PONTE), Administrative Studies, Faculty of Management and Business Tampere University Tampere Finland; ^2^ Department of Biology University of Turku Turku Finland; ^3^ Department of Conservation Biology & Social‐Ecological Systems Helmholtz Centre for Environmental Research UFZ Leipzig Germany; ^4^ Social Sciences LUT School of Engineering Sciences, LUT University Lappeenranta Finland; ^5^ FinEst Centre for Smart Cities Tallinn University of Technology Tallinn Estonia; ^6^ Finnish Environment Institute Helsinki Finland; ^7^ Finnish Museum of Natural History University of Helsinki Helsinki Finland; ^8^ The Association for Ecological Forestry Certification Ry Helsinki Finland

**Keywords:** biodiversity policy, conservation actions, conservation interventions, leverage points, mainstreaming, paradigms, Red List of Ecosystems, Integración, intervenciones de conservación, lista roja de ecosistemas, medidas de conservación, paradigmas, política de biodiversidad, puntos de influencia, 生物多样性政策, 保护行动, 保护干预措施, 杠杆点, 主流化, 范式, 生态系统红色名录

## Abstract

Red lists provide critical knowledge regarding biodiversity decline, especially in Finland, where broad assessments have been made regularly since the 1980s. They deliver information on the threat status of species and ecosystems, propose actions to guide conservation policy, and have the potential to spur transformative change. We examined whether the transformative potential of the proposals has changed over time. We analyzed the contents qualitatively and quantitatively of seven Finnish Red Lists of Species or Ecosystems from 1986 to 2019. We used a prior analysis of transformative potential of conservation actions in the Conservation Measures Partnership classification (such as outreach or conservation designation and planning), which identified Meadows’ sustainability leverage points (i.e., in complex systems, a point at which a small change can lead to large changes) associated with each action category. We also determined the number of proposals that overlapped with sectors beyond conservation. We used a nonparametric Mann–Kendall trend test and linear models to analyze temporal trends in the data. Cross‐sectoral proposals increased over time, but there was only a small shift toward actions that influenced the root causes of biodiversity loss (i.e., deep leverage actions). In the qualitative assessment of how the actions were proposed to be implemented, actions became more complex and effectiveness of implementation increased, demonstrating a change within conservation action categories toward deeper leverage points. This increasing transformative potential can be a catalyst and a consequence of broader societal change driven by ongoing biodiversity loss. Red lists could play a role in transformative change, but the engagement of actors across society in devising action proposals must be inclusive and cover expertise from the social sciences and humanities. Our results emphasize the importance of considering complementary dimensions of transformative change simultaneously to achieve a comprehensive understanding of viable paths to societal change.

## Abstract (es)

Un marco para maximizar los beneficios de la conservación de la regeneración forestal en terrenos privados

**Resumen:**

Las listas rojas aportan conocimientos fundamentales sobre la disminución de la biodiversidad, especialmente en Finlandia, donde se llevan a cabo evaluaciones exhaustivas de forma regular desde la década de 1980. Proporcionan información sobre el estado de amenaza de las especies y los ecosistemas, proponen medidas para orientar las políticas de conservación y tienen el potencial de impulsar un cambio transformador. Examinamos si el potencial transformador de las propuestas ha cambiado con el tiempo. Analizamos cualitativa y cuantitativamente el contenido de siete Listas Rojas finlandesas de especies o ecosistemas desde 1986 hasta 2019. Utilizamos un análisis previo del potencial transformador de las acciones de conservación en la clasificación de la Conservation Measures Partnership (como la divulgación o la designación y planificación de áreas de conservación), que identificó los puntos de palanca de sostenibilidad de Meadows (es decir, en sistemas complejos, un punto en el que un pequeño cambio puede conducir a grandes cambios) asociados a cada categoría de acción. También determinamos el número de propuestas que se solapaban con sectores más allá de la conservación. Utilizamos una prueba de tendencia no paramétrica de Mann‐Kendall y modelos lineales para analizar las tendencias temporales de los datos. Las propuestas intersectoriales aumentaron con el tiempo, pero solo se observó un ligero cambio hacia acciones que influyeran en las causas fundamentales de la pérdida de biodiversidad (es decir, acciones de gran impacto). En la evaluación cualitativa de cómo se proponía implementar las acciones, estas se volvieron más complejas y la eficacia de su implementación aumentó, lo que demuestra un cambio dentro de las categorías de acciones de conservación hacia puntos de mayor impacto. Este creciente potencial transformador puede ser un catalizador y una consecuencia de un cambio social más amplio impulsado por la pérdida de biodiversidad en curso. Las listas rojas podrían desempeñar un papel en el cambio transformador, pero la participación de los actores de toda la sociedad en la elaboración de propuestas de acción debe ser inclusiva y abarcar los conocimientos especializados de las ciencias sociales y las humanidades. Nuestros resultados ponen de relieve la importancia de considerar simultáneamente las dimensiones complementarias del cambio transformador para lograr una comprensión integral de las vías viables hacia el cambio social.

## 1986年至2019年间芬兰红色名录中行动建议的变革潜力变化

**【摘要】** 红色名录为生物多样性丧失提供了关键信息, 尤其是在芬兰, 该国自20世纪80年代以来便定期开展广泛评估。这些名录不仅提供了物种和生态系统的受威胁状况信息, 还提出了指导保护政策的行动建议, 并有潜力推动转型变革。本研究分析了这些建议的变革潜力是否随时间推移而发生变化。我们对1986年至2019年间七份芬兰物种或生态系统红色名录的内容进行了定性与定量分析。研究利用了“保护措施伙伴关系”分类体系中关于保护行动变革潜力的前期分析(如公众宣传或保护区划定与规划), 该分析识别了与每个行动类别相关的梅多斯(Meadows)可持续性杠杆点(即在复杂系统中, 微小变化可引发巨大变化的临界点)。此外, 我们还统计了与保护领域以外部门存在重叠的建议数量。研究进一步利用非参数Mann–Kendall趋势检验和线性模型分析了数据的时间趋势。我们发现, 跨部门的建议随时间推移有所增加, 但针对影响生物多样性丧失根本原因的行动(即深层杠杆行动)仅出现微小转变。在对行动拟定实施方式的定性评估中, 行动变得更加复杂, 实施有效性有所提高, 表明保护行动类别内部正朝着更深层的杠杆点转变。这种日益增强的变革潜力既可能是生物多样性持续丧失所驱动的更广泛社会变革的催化剂, 也可能是其结果。红色名录可在转型变革中发挥作用, 但全社会各利益相关方在参与制定行动建议时必须具有包容性, 并涵盖社会科学和人文学科的专业知识。我们的研究结果强调, 必须同时考虑转型变革的互补维度, 以全面理解实现社会变革的可行路径。**【翻译:胡怡思;审校:聂永刚】**

## INTRODUCTION

Transformative change is needed to halt biodiversity decline (IPBES, 2019). Despite decades of international treaties and biodiversity goals and increasing awareness of biodiversity loss and climate change, current development pathways remain largely unchanged (IPBES, 2019). The Intergovernmental Science–Policy Platform on Biodiversity and Ecosystem Services (IPBES) defines transformative change as “a fundamental, system‐wide reorganization across technological, economic, and social factors, including paradigms, goals, and values” (IPBES, 2019). Yet, actors have rarely assessed conservation policies and actions from the perspective of transformative change until recently (Arponen & Salomaa, 2023; Bulkeley et al., 2020; Davila et al., 2021; Pascual et al., 2022; Salomaa & Arponen, 2023; Wittmer et al., 2021). Understanding transformative change is a high priority of the global biodiversity policy community, addressed by, for example, the IPBES in their extensive Transformative Change Assessment (IPBES, 2024) and the Global Biodiversity Framework. A historical perspective on the transformative potential of biodiversity policies can provide valuable insight into the underlying reasons for failure or success.

There have been few attempts to empirically study transformative change—interlinked, cascading changes across sectors—from the cross‐sectoral perspective (Salomaa & Juhola, 2020). Cross‐sectorality refers to the interaction and collaboration between different sectors of society—in our case, between nature conservation and other sectors (e.g., agriculture, forestry, regional planning, and construction). Mainstreaming of biodiversity across sectors throughout society (also known as policy integration across governance sectors; Pröbstl et al., 2023) has been proposed as a solution for halting biodiversity decline. In 2018, the Fourteenth Conference of the Parties of the Convention on Biological Diversity (CBD) established a long‐term strategic approach to mainstreaming (CBD, 2018). Relevant literature recognizes integration across scales, places, issues, and sectors as a key requirement for transformative governance, together with inclusivity of rights holders, knowledge holders, and stakeholders (Pascual et al., 2022; Visseren‐Hamakers et al., 2021). Yet, mainstreaming has been identified as a major challenge in the implementation of the CBD through national biodiversity strategies and action plans (Cardona Santos et al., 2023; Whitehorn et al., 2019) and has taken place in cycles subject to political dynamics instead of a steady increase (Reber et al., 2022).

One possible reason for the weak effectiveness of mainstreaming is that changes in deep leverage points (i.e., in complex systems, a point at which a small change can lead to large changes), which touch on the goals and paradigms of systems, are inherently challenging (Meadows, 1999). Termeer et al. (2024) identified three dimensions of transformative change—quick, in depth, and system wide—but claim that not all can be achieved simultaneously due to inherent trade‐offs. The in‐depth dimension can be described through leverage points, and system‐wide approaches can be analyzed via cross‐sectorality.

Meadows (1999) originally described leverage points as a framework for systems analysis, although since then, there has been mixed use of the concept (Leventon, Abson, et al., 2021). Koskimäki (2021) described Meadows’ leverage points as “key *system properties* where focused interventions can give rise to large changes in the behavior of a system.” Meadows’ (1999) leverage points framework did not explicitly consider cross‐sectorality. Nonetheless, researchers have studied multiple sectors as part of one system and compared several systems (Fischer et al., 2022). Further, researchers have noted the importance of understanding the nestedness of systems and the connections of the study system to other systems (Leventon, Abson, et al., 2021) and interactions among leverage points (Abson et al., 2017; Dorninger et al., 2020) for advancing transformative change. Hartel et al. (2019) identified leverage points for mainstreaming interventions, and Chan et al. (2020) viewed coordination across sectors and jurisdictions as a lever in their framework. However, the approach used by Chan et al. (2020) differs from that of Meadows (1999) in that their leverage points are closer to system outcomes than properties (Koskimäki 2021). Scott et al. (2022) linked deeper leverage interventions with stronger mainstreaming, acknowledging their emphasis on collaborative work, coproduction, and knowledge exchange. Indeed, interpretations of the relationship between leverage points and cross‐sectorality vary, but it is evident that cross‐sectorality is not fully captured by the leverage points framework. Instead, the approaches complement each other, aligning with Termeer et al.’s (2024) system‐wide and in‐depth dimensions of transformative change.

Biodiversity assessments contribute to shaping biodiversity policy. Recently, there have been calls for policy‐prescriptive or solution‐oriented environmental assessments to advance transformative change (Beck et al., 2022). Red lists (RLs) are biodiversity assessments that provide information on the numbers of threatened species and ecosystems in an area, often a nation (IUCN, 2012). RLs are used to guide national legislation and various policies in many countries (Bland et al., 2019; Botts et al., 2020). They can potentially contribute to public policies (Alaniz et al., 2019) and the implementation of the Global Biodiversity Framework (Nicholson et al., 2024) in a number of different ways, but they have not yet been assessed from the perspective of impacting transformative change. Generally, RLs provide one perspective on biodiversity loss, but they are challenging to harness directly for policy and practice. The need for a tool summarizing their results led to the development of the International Union for Conservation of Nature (IUCN) Red List Index, which shows trends in the overall conservation status of sets of species as an indicator of trends in biodiversity (Bubb et al., 2009; Butchart et al., 2007). RL indices are used, for example, to monitor extinction risk in different ecosystems at national scales (Juslén et al., 2013, 2016; Raimondo et al., 2023). However, even RL indices do not specifically address the question of what should be done to reverse these negative trends. In Finland, RL assessments have always contained concrete proposals for action that are devised based on the threat assessment (e.g., Hyvärinen et al., 2019; Kontula & Raunio, 2018). Australia, Norway, and Sweden have also devised recommendations for action based on RL classifications (Eide et al., 2020; Kyrkjeeide et al., 2021; Walsh et al., 2013), but Finnish action proposals provide a unique chance to study a four‐decade‐long historical perspective. We assessed the development of transformative change at the Finnish science–policy interface and the extent to which the proposals reflect the development of conservation science and the broader development of conservation policy and practice.

We sought, first, to develop an analytical approach based on Meadows’ (1999) leverage point framework and cross‐sectorality that is applicable to assessing the transformative potential of any policy, strategy, or other document that describes conservation actions. Second, we sought to provide a historical perspective on the contribution of RLs to transformative change by analyzing, to our knowledge for the first time, RL action proposals. We hypothesized that increasing scientific understanding of the socioecological complexity of biodiversity loss is connected with increasing cross‐sectorality and deeper leverage in action proposals. Our research questions were do leverage points associated with action proposals become deeper over time, do cross‐sectorality and the complexity of action proposals increase over time, and does the composition of action proposals change. We assessed these questions quantitatively across action categories and qualitatively within action categories concerning their proposed implementation.

## METHODS

We considered all cross‐taxon RL assessments published in Finland up to 2019, which included two species assessments (Rassi et al., 1986, 1992) done following national criteria prior to the publication of the assessment protocol of the IUCN (2001) and three species assessments following the IUCN protocol (Hyvärinen et al., 2019; Rassi et al., 1986, 1992). There were also two assessments of ecosystems (Kontula & Raunio, 2018; Raunio et al., 2008). The latter ecosystem assessment followed the IUCN protocol for the Red List of Ecosystems (RLE) (Bland et al., 2017), and the former predated the existence of the protocol. We refer to both as RLEs for simplicity. Pre‐ and post‐IUCN protocol assessments have different criteria for classifications, and the results are not directly comparable. However, the action proposal sections are not dependent on the criteria used. The latest species assessment (Hyvärinen et al., 2019) contained a section on action proposals but referred to a previously published national action plan for species conservation (Ministry of the Environment, 2017) as a more comprehensive source of action proposals that the assessment fully supported. Thus, we merged the proposals from the action plan with the 2019 assessment. All assessments were remarkably extensive. Coverage of species assessments increased from 370 vertebrates and altogether nearly 15,000 species in 1986 to 379 vertebrates and 22,418 species in 2019, amounting to 47% of known species in Finland. The RLEs covered 368 and 388 ecosystems at the most detailed levels of classification in 2008 and 2018. Details about the expert groups conducting the assessments can be found in Appendix S1.

### Study system

Our study system is the Finnish social–ecological system, with a special focus on the knowledge system (RLs) and biodiversity conservation. Finland is a sparsely populated Nordic country largely covered by boreal forests (ca. 75%; Forest News, 2025), scattered by peatlands and numerous lakes. In 2019, 11.9% of the assessed species were threatened, mainly by forest management activities, the overgrowing of open ecosystems, and changes in arable land use (Hyvärinen et al., 2019). Of ecosystems, 48% were assessed as threatened; the most threatened ones occur among traditional rural biotopes, forests, and wetlands (Kontula & Raunio, 2018).

### Analytical framework

**FIGURE 1:**
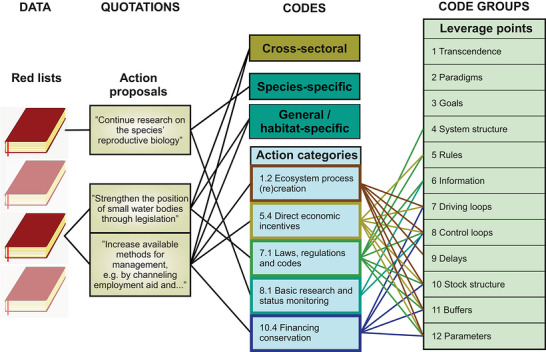
Overall analysis structure and coding hierarchy in Atlas.ti used to analyze the contents of the red lists. Action proposals from the Finnish Red Lists are coded as species specific or general or ecosystem specific (general proposals and ecosystem‐specific proposals are pooled because they resembled each other). They can contain multiple action categories and include cross‐sectoral coding. An action category can be associated with multiple leverage points (i.e., point at which a small change can lead to large changes). Individual action proposals can be linked to a leverage point through multiple pathways. A full list of action categories is in Table 1, and descriptions of leverage points with examples of actions are in Table 2. The full list of action categories and their associations with the leverage points is in Appendix S2.

Figure 1 shows our analysis structure for the transformative potential of conservation actions. The body of our analytical framework was a combination of two categorizations: the Conservation Actions Classification 2.0 of the Conservation Measures Partnership (2016) (Table 1) and the leverage points framework (Meadows, 1999) (Table 2). We used Arponen and Salomaa's (2023) assessment of conservation actions, which links each action to its potential leverage points (Table 2) (a full table of associations is in Appendix S2), to determine the transformative potential of each action. Meadows’ (1999) leverage points have been used in the past for qualitative coding in diverse contexts (Carey & Crammond, 2015; Dorninger et al., 2020; Lidgren et al., 2006; Manlosa et al., 2019; Rosengren et al., 2020). By definition, the transformative potential of an action implies the action may not deliver the desired effect for many reasons, and an action category may contain heterogeneous actions with varying leverage points (e.g., outreach varies from press releases to immersive stakeholder engagement methods). Leverage points vary from shallow (i.e., those that treat symptoms without changing the system) to deep (defined above). Shallow leverage points are easier to influence but have a weaker impact on the system as a whole. A typical example would be site stewardship measures (Action Category 1.1). Deep leverage points may take time and effort to reach, but they address the root causes of biodiversity decline through, for example, outreach that reinforces nature connectedness (Arponen & Salomaa, 2023).

**TABLE 1:** Action categories from conservation action classification of the Conservation Measures Partnership.

A. Target restoration or stress reduction		
	1. Land or water management	1.1 Site or area stewardship 1.2 Ecosystem and natural process (re)creation
	2. Species management	2.1 Species stewardship 2.2 Species reintroduction and translocation 2.3 Ex situ conservation
B. Behavioral change or threat reduction		
	3. Awareness raising	3.1 Outreach and communications
	4. Law enforcement and prosecution	4.1 Detection and arrest 4.2 Criminal prosecution and conviction 4.3 Nuoncriminal legal action
	5. Livelihood, economic, and moral incentives	5.1 Linked enterprises and alternative livelihoods 5.2 Better products and management practices 5.3 Market‐based incentives 5.4 Direct economic incentives 5.5 Nonmonetary values
C. Enabling condition		
	6. Conservation designation and planning	6.1 Protected area designation and/or acquisition 6.3 Land‐ or water‐use zoning and designation 6.4 Conservation planning
	7. Legal and policy frameworks	7.1 Laws, regulations, and codes 7.2 Policies and guidelines
	8. Research and monitoring	8.1 Basic research and status monitoring 8.2 Evaluation, effectiveness measures, and learning
	9. Education and training	9.1 Formal education 9.2 Training and individual capacity development
	10. Institutional development	10.1 Internal organizational management and administration 10.2 External organizational development and support 10.3 Alliance and partnership development 10.4 Financing conservation

*Note*: Only the categories for which we had data are listed.

**TABLE 2:** Meadows’ (1999) conceptual framework of leverage points for sustainability and practical examples of it in the context of conservation actions from Arponen and Salomaa (2023).

Leverage point (decreasing order of effectiveness, from deep to shallow leverage)^a^	Example (Arponen & Salomaa, 2023)
1. Power to transcend paradigms (transcendence)	Well‐designed outreach (3.1) through transdisciplinary, participatory approaches could enlighten the target audience regarding coexistence and value of worldviews beyond their own, inducing transcending paradigms.
2. Mindset or paradigm out of which the system—its goals, structure, rules, delays, and parameters—arises (paradigms)	Alliance and partnership development (10.3) can change paradigms to reconstruct power relations, build relationships, and change mental models.
3. Goals of the system (goals)	Outreach (3.1) that reinforces nature connectedness also has the potential to influence the system paradigm and goals.
4. Power to add, change, evolve, or self‐organize system structure (system structure)	Providing a more diverse set of alternatives for livelihoods (5.1) could give room for the system structure to self‐organize.
5. Rules of the system (rules)	Environmental markets (5.3) represent new rules, as do certification schemes at a different level.
6. Structure of information flows (information)	Formal education (9.1) addresses information flows.
7. Gain around driving positive feedback loops (driving loops)	Outreach (3.1) concerning conservation success stories that have brought benefits to the local community can encourage more active participation, forming a reinforcing feedback loop around the conservation action itself.
8. Strength of negative feedback loops relative to the impacts they are trying to correct (control loops)	Environmental markets (5.3, carbon markets, ecological compensation schemes) would provide controlling feedback loops through internalizing costs.
9. Length of delays relative to the rate of system change (delays)	Actively restoring (1.2) instead of letting nature take its course reduces delays in recovery (e.g., when infilling ditches in drained peatlands instead of just stopping their maintenance).
10. Structure of material stocks and flows (stock structure)	Structure is critical for creating processes (1.2), from structural components, such as retention trees, to overall age structure of a forest or spatial connectivity of an ecosystem.
11. Sizes of buffers and other stabilizing stocks relative to their flows (buffers)	Maintaining viable populations of species in captivity (2.3) and gene banking represent a type of buffering approach by definition.
12. Constants, parameters, numbers (parameters)	Site or area stewardship (1.1) includes small‐scale actions with a physical character; enhancing viability or mitigating stress implies changing a quantity.

*Note*: Short names for the leverage points used in Figure 1 are in parentheses.

^a^ Definitions: deep leverage points influence root causes of biodiversity loss; shallow leverage points treat symptoms without changing the system.

We considered proposals that covered sectors outside the core conservation realm (e.g., agriculture, forestry, regional planning, or construction) as cross‐sectoral (Figure 1). Our definition of *cross‐sectoral* included any activity that reached beyond the borders of the environmental sector in any way, ranging from administration across ministries to engagement of stakeholders across sectors. Due to our focus on change, we did not consider hunting cross‐sectoral because of the long, shared history of conservation and the population management aspect of hunting in Finland (Vuorisalo & Laihonen, 2000) and because of hunters’ long‐term participation in wildlife monitoring (Helle et al., 2016).

### Coding

We used Atlas.ti 23 software for qualitative data analyses. It enables a hierarchical coding structure well suited for our analysis framework, which had the lower level of action categories (codes) and higher level of leverage points (code groups) (Figure 1; Table 2; Appendix S2). We also coded the contents according to the sections of RLs. Action proposals in species‐specific text sections were coded as species specific, and proposals in separate proposal chapters and those in RLEs were coded as general proposals. When proposed actions were directly linked with other actions, we coded them under each, as recommended by Arponen and Salomaa (2023). For example, a law proposal (7.1) to provide economic support (5.4) for the management of herb‐rich forests (1.1) was coded under all three categories because we wanted to distinguish between different kinds of laws and make visible chains of leverage and their expected impacts. We checked the quotations code by code in a second round of coding. The final choices for ambiguous proposals were based on discussions among all coauthors (Appendix S1).

### Analyses

We quantified the trends in the proportional shares of action proposals per action category and in the leverage points linked to the action proposals over time. We used a nonparametric Mann–Kendall trend test to check for trends in individual action proposals or leverage points and fitted linear models to test for the scale of overall temporal change in the proportional shares of action proposals. We also fitted linear models to test for trends in the number of codings (action categories) per quotation from the RL as an indicator of proposal complexity and interactions among actions and in the cross‐sectorality of action proposals. We performed all analyses with R 4.4.1 (R Core Team, 2024). We used the Kendall package 2.2.1 (McLeod, 2022) for the Mann–Kendall trend tests. For details, see Appendix S3.

We also qualitatively assessed the proposed actions for temporal trends within each action category and noted actions that were persistently emphasized across the study period. Where quantitative results are not referred to, the results are based on a qualitative assessment.

## RESULTS

**FIGURE 2:**
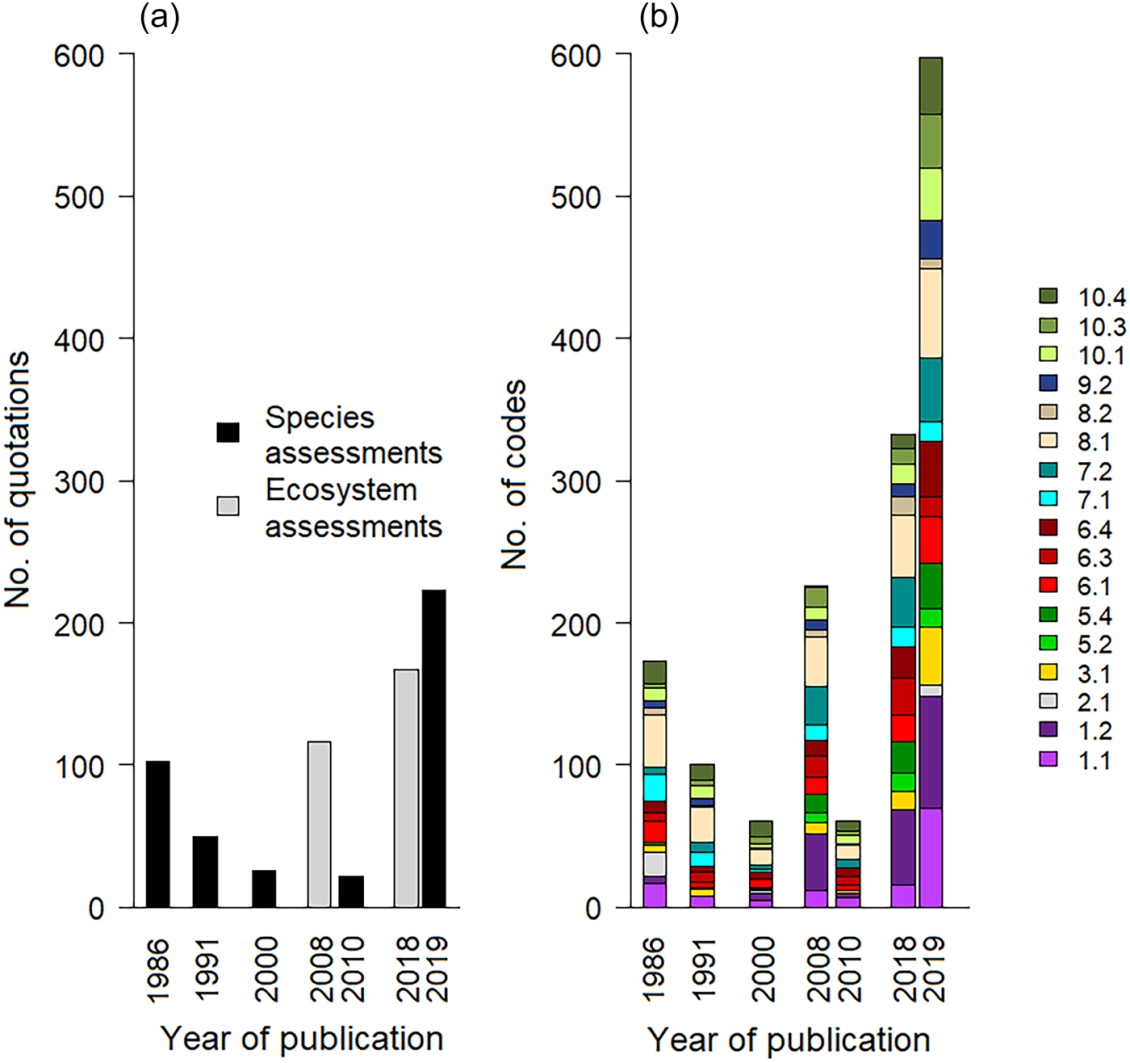
Numbers of (a) general (i.e., not species specific) conservation action proposals and (b) numbers and types (colors) of action category codes (Table 1) across seven Finnish Red Lists (1986–2019) (details in Appendix S3). For readability, in (b) results are for the action categories with ≥5% share of the total number of action proposals in at least 1 red list (1551 action proposals) (numerical results in Appendix S15).

We coded 2289 action proposals as separate quotations in the dataset, which were associated with 4448 action category codings. The species‐specific action proposals were strongly biased toward the 1986 assessment (54%), and the distribution of action categories also differed from the general proposals (Appendix S4). As our main question was to examine temporal trends, we decided to focus on the general proposals that formed a more coherent dataset (proposals in general sections of species assessments and ecosystem‐specific proposals [hereafter, *proposals*]; total 706 action proposals and 1613 action codes) and to present the results for the species‐specific proposals in Appendices S4–S14 and S19–S22 only. For simplicity, we did not include in the body of the article action categories that did not reach 5% of the total action proposals for any year (10 action categories) (Appendix S15), leaving us with 1551 coded action proposals (Figure 2).

### Quantitative results for leverage points and cross‐sectorality

**FIGURE 3:**
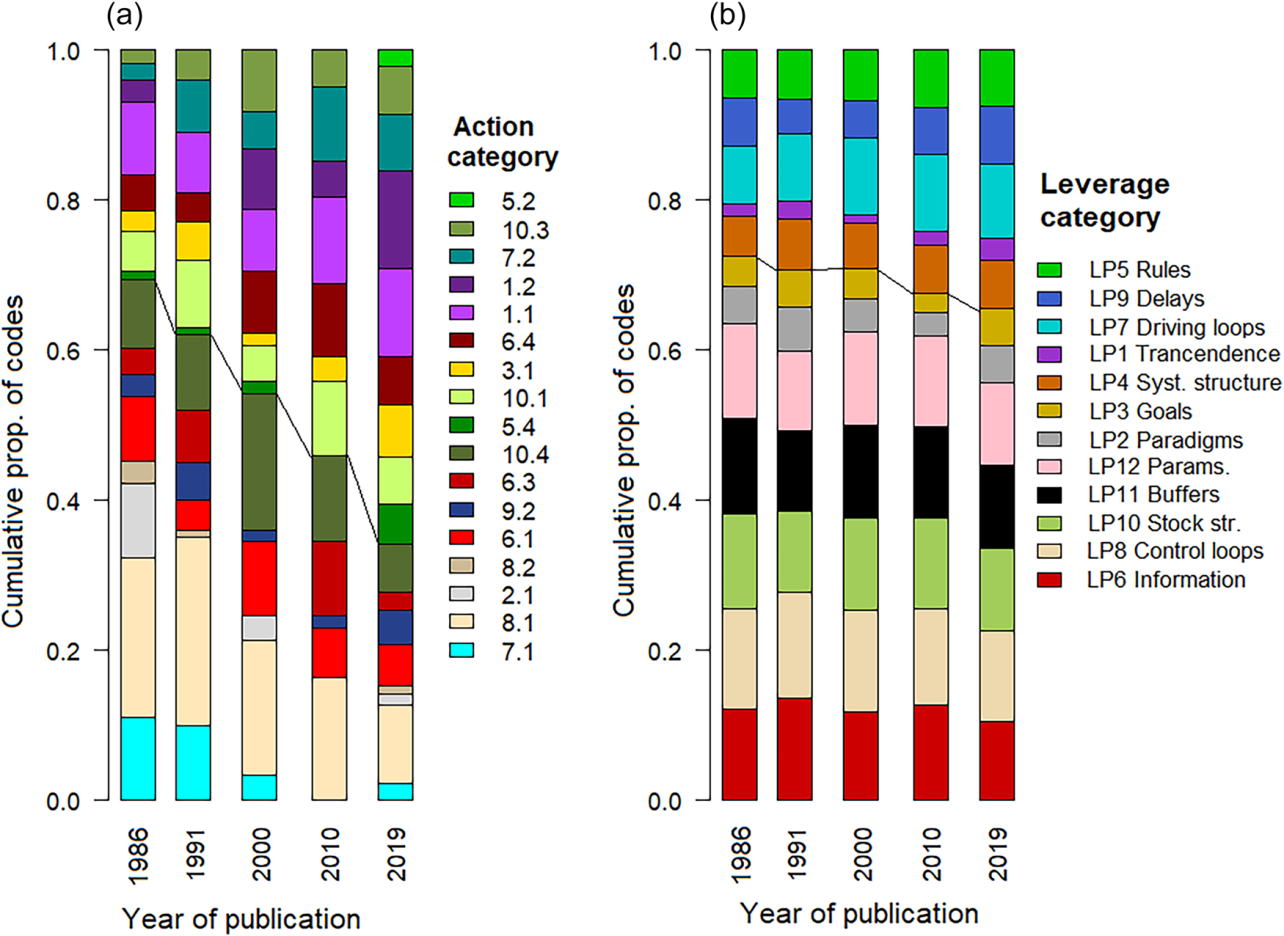
Proportion of (a) actions and (b) leverage points of general (i.e., not species specific) conservation action proposals in the five Finnish Red Lists of species by the year of publication and sorted in decreasing order within each year by Kendall's tau of the category. The black line shows the change in cumulative proportion of nonincreasing categories over time (range 0.71–0.35 for action and 0.73–0.65 for leverage categories) (details in Appendices S3 & S6). For readability, results are shown only for action categories with ≥5% share of the total number of action proposals per year in at least 1 red list (1551 action proposals) (Appendix S15).

In our quantitative analysis, all leverage points were included in all assessments. The quantitative data lacked a clear trend toward deeper leverage over time. However, the cumulative share of the seven nonincreasing (i.e., Kendall's τ ⪬ 0.5) (Appendix S3) leverage point associations decreased from 72.5% to 65.4% (*p* < 0.001, *F*_1,3_ = 55.09, *R* ^2^ = 0.9168) (Figure 3), indicating a statistically significant but small change over time (Appendix S3). Roughly 70–85% of proposals addressed at least one of the shallowest parameter leverage points (10–12), and the percentage of proposals reaching the deep leverage points (1–3) related to paradigms and goals of the system ranged from 10–40% (Appendix S16). Rules (leverage point 5) were addressed in the postmillennial general proposals more frequently than before (*p* < 0.05) (Appendix S8).

**FIGURE 4:**
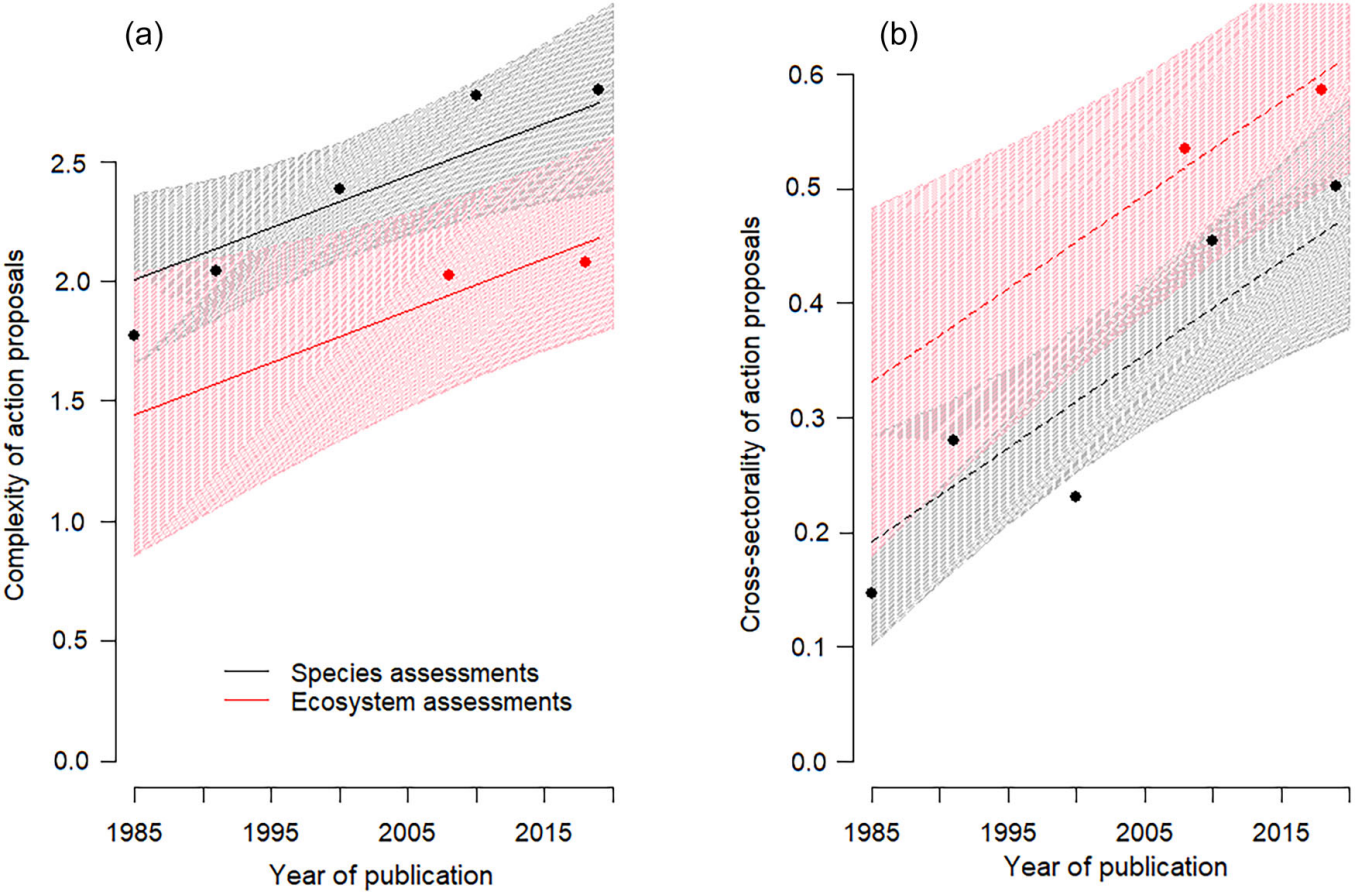
(a) Complexity and (b) cross‐sectoral scores (filled circles) of conservation action proposals in seven Finnish Red Lists for two types of assessments over time (lines, fitted linear model; shading, 95% confidence intervals). These models include the species‐specific action proposals that were omitted from the “RESULTS” section (full results in Appendices S3–S14).

There was an increasing temporal trend in the cross‐sectorality of the action proposals (*R* ^2^ = 0.934, *F*_3,7_ = 33.1, *p* < 0.001) (Figure 4; Appendix S11). In 1986 only 15% of the proposals were cross‐sectoral, whereas in all postmillennial assessments, approximately half were cross‐sectoral, being highest in the RLEs (Appendix S15, second‐to‐last row). Cross‐sectorality was self‐evidently associated with land‐use zoning, as well as with livelihood and economic incentive actions, which were concentrated in the postmillennial assessments. However, cross‐sectorality increased over time in the majority of the action categories (Appendix S17). It was also visible in conventional conservation actions, for example, in the 2010 recommendation to focus conservation programs of species under strict protection to “the most endangered species only, the conservation of which involves several parties and complicated issues, and for which the programs are therefore necessary.”

Action proposals also became more complex over time (*R* ^2^ = 0.837, *F*_3,7_ = 12.0, *p*< 0.01) (Figure 4), addressing multiple actions. The mean number of action codings per proposal increased from 1.92 to 3.3 (Appendix S15). For example, where the earlier assessments would provide recommendations to protect a valuable site, more recent assessments would give guidance on available funding instruments, division of responsibilities, and outreach to stakeholders to more effectively maintain and restore ecosystem quality.

### Quantitative results for the action proposals

The composition of the proposed actions changed over time in the species assessments. The cumulative share of the 14 nonincreasing action proposal categories decreased from 70.7% to 35.1% over time (*p* < 0.001, *F*_1,3_ = 344.1, *R* ^2^ = 0.9914) (Figure 3). Basic research and status monitoring (8.1) was the most frequent action category in the full dataset (32% of quotations) and consistently among the top three categories across all assessments (Figure 2; Appendix S15). Ecosystem and natural process creation or re‐creation (1.2), alliance and partnership development (10.3), and different incentives (5.1–5.5) increased over time (Figure 2; Appendix S6). Ecosystem assessments were excluded from the quantitative analyses of temporal trends because they differed from species assessments in terms of their action category and associated leverage point composition and there were too few (two) points for a separate analysis.

### Land, water, and species management actions

Land and water management actions were among the most frequent action proposals across assessments (Actions 1.1–1.2 in Appendix S15). Ecosystem and natural process creation or re‐creation (1.2) rapidly grew from a minor category to the largest one in 2019. The emphasis changed from identifying which site management actions were needed to improving their implementation (see also Appendix S18). The proposals increasingly discussed spatial planning, cost effectiveness, new policy instruments (e.g., ecological compensation), funding sources, stakeholder and community engagement, organizational management issues, responsibilities, cross‐sectoral collaboration, and monitoring of their effectiveness. The majority of these aspects help reach deeper leverage points than these actions would reach alone (Appendix S2). Species management actions operate mainly at shallow leverage points (leverage points 9–12) (Appendix S2), but, here too, the trend was toward broader proposals regarding, for instance, their funding and organization.

### Outreach

The percentage of proposals with an outreach aspect was fairly constant but peaked in 2019 at 18% (Figure 3; Appendix S15). The quantitative assessment gave credit to all outreach activities in representing all leverage points (Appendix S2), but the qualitative assessment again revealed some temporal trends toward deeper leverage. The early assessments focused on spreading information about threatened species to the general public and engaging nature enthusiasts in collecting data (both aspects linked with research 8.1 [Appendix S18]). Later assessments emphasized outreach to different stakeholders and suggested professional counseling for various stakeholders whose activities have an impact on biodiversity, especially regarding alternative management practices, novel policies, and opportunities for conservation. Encouraging the general public to participate in voluntary activities and take care of their local environment (e.g., through the removal of invasives) was a rising trend with potentially deep leverage impacts.

### Livelihood and economic incentives

Apart from one proposal in 1986 to develop osprey‐friendly deterrent devices for fish farms, the better products and management practices (5.2) category appeared only starting in 2008. Although these actions are of shallower leverage than other types of incentives, these proposals were all cross‐sectoral. Direct economic incentives (5.4) were more common in the postmillennial assessments, and their purposes and sources became more diverse over time, for example, with the appearance of EU agricultural subsidies and new voluntary policies. Since 2008, suggestions to develop new incentives to support entrepreneurial activities and innovation have emerged. Their focus extends beyond the context of agriculture and forestry (e.g., investigating the potential for ecological compensation).

### Conservation designation and planning

Protected area designation or acquisition (6.1) was consistently proposed across the assessments. The first two assessments addressed the development and implementation of national conservation programs underway at that time. Recommendations to increase the area protected received attention throughout the assessments, but the postmillennial proposals became more diversified, addressing, for example, protected area management and restoration, adequacy of funding, and incentives for establishing voluntary protected areas. These were often coupled with proposals regarding conservation planning (6.4) in which cost‐effective modeling and spatial prioritization, remote data, cross‐sectoral stakeholder collaboration, and the training of planners became salient over time (see also Appendix S18). Therefore, even though conservation planning in isolation is considered to influence information flows only (leverage point 6), the associated actions showed a trend toward deeper leverage and cross‐sectorality. Similar trends were observed in the land‐ or water‐use zoning and designation (6.3) proposals.

### Laws and policies

Laws, regulations, and codes (7.1) had a decreasing trend over time (Appendix S6). The assessment in 1986 addressed the revision of the Nature Conservation Act that had been underway since the 1960s, making many detailed proposals regarding its contents. In 1986, several acts regarding forestry were also addressed, and the 1992 assessment mentioned that all laws regarding the use of natural resources and land use should consider conservation and sustainability. Over time, the focus shifted from action proposals for restrictions, quotas, and licensing to cross‐sectoral issues, such as pollution, construction, infrastructure, transport, and agrienvironment scheme development. These proposals addressed drivers of biodiversity loss and thus corresponded to deeper leverage points than, for instance, regulation regarding a specific protected area. In addition, proposals for impact evaluation of legal frameworks and proposals to simplify regulations that delay conservation emerged in the latest assessments. Proposals for policies and guidelines (7.2) were more heterogeneous than law codings but had a similar overall trend toward increasing cross‐sectorality.

### Research and monitoring

Basic research and status monitoring (8.1) was considered in 26–50% of proposals each year (Figure 3; Appendix S15). Constant worries concerned the lack of data on species, ecosystems, and the effectiveness of conservation actions, the need for continuous monitoring, and securing future RL assessments. Over time, more diverse and timely research questions were identified for basic research, as well as evaluation, effectiveness measures, and learning (8.2). Rising topics since 2008 concerned, for example, diverse human impacts on biodiversity and the effectiveness of various solutions, such as financial incentives. Development activities and piloting were in a growing role, including research on ecological compensation. There were specific recommendations to extend the funding for research on deficiently known and threatened species and ecosystems and to take advantage of new methods, such as remote sensing or spatial prioritization.

Organizational management actions regarding coordination and responsibilities for research became more frequent over time, as did cross‐sectoral collaboration in research and knowledge sharing (Appendix S18). The need for a biodiversity data repository was already recognized in 1986, but action proposals became more frequent and detailed over time, emphasizing knowledge availability to all stakeholders and, since 2017, addressing the development of the Finnish Biodiversity Information Facility (FinBIF) (established in 2017).

### Individual and institutional capacity building

Training and individual capacity development (9.2) proposals first emphasized the training of public authorities and nature enthusiasts regarding conservation management and species identification. These were later accompanied by largely cross‐sectoral action proposals (Appendix S17) regarding training for the use of the newly opened data sources, as well as counseling and capacity‐building for stakeholders. Internal organizational management (10.1) proposals concerned the first environmental administration and research organizations. The postmillennial assessments dedicated attention to data availability and the division of responsibilities across organizations. Alliance and partnership development (10.3) was suggested increasingly cross‐sectorally (Appendix S17) and internationally, and it was seen to have a key role in developing and running the biodiversity data repository. Action proposals about financing conservation (10.4) were always linked to other actions to be funded, and the trends therefore reflected the general trends described above. These were almost exclusively about the distribution of public funds, apart from 2019, when the possibilities for the participation of private businesses were briefly recognized.

## DISCUSSION

Our most prominent observation was that the cross‐sectorality and complexity of action proposals increased dramatically over time. In the qualitative assessment, we found changes in the ways the actions were proposed. More complex ensembles of actions in the recent assessments addressed how to implement actions more effectively with different objects of action, reaching deeper leverage points and broader cross‐sectorality. The quantitative leverage points analysis did not capture many significant temporal trends because the most frequent action categories remained relatively stable over time, and because most actions were associated with several leverage points—any changes that did take place blended into the large mass of shallow leverage point associations.

With respect to the three governance pathways of Termeer et al. (2024), it appears that system wide was the easiest dimension to advance in the context of Finnish RLs, but it would require stronger engagement of an additional dimension to achieve one of Termeer et al.’s (2024) suggested pathways for transformative governance: the “rule change” pathway through system wide and quick change or the “big plans” pathway through system wide and in‐depth dimensions. The observed increase in cross‐sectorality aligns well with, for example, the CBD goals and the present scientific understanding of how to address the wicked problems driving biodiversity loss. The cross‐sectoral codings address not only a governance structure perspective but also the increasing importance of partnerships and collaborations and engaging different stakeholders, representing the plurality and inclusiveness that are characteristic to much of the transformations and transitions literature (Bodin, 2017; Chambers et al., 2022; Leventon, Duşe, et al. 2021; Miller & Wyborn, 2020; Pascual et al., 2021; Zafra‐Calvo et al., 2020).

### Potential drivers behind the observed changes

Disentangling a genuine transformative shift in thinking from other impacts on the proposed actions is challenging. Some trends may have been influenced by grasping the low‐hanging fruit in the early stages; hence, remaining actions could be inherently more complex, rather than reflecting an intentional shift in strategy. The results were clearly partly driven by trends reflecting current, sometimes even singular, events in policy and research, which may or may not be part of a broader transformative paradigm shift. These include, for example, changes in environmental administration structure (Ministry of the Environment, 2007) and active development and adoption of spatial conservation prioritization methods in decision making in Finland (Moilanen et al., 2019). Diverse new national and EU legislation was implemented over the study period (Sairinen, 2003, p. 200). Apart from new agricultural policies visible in proposals concerning subsidies and the counseling of landowners, EU legislation was not frequently mentioned in the proposal. Instead, the assessment in 1986 gave extensive recommendations regarding the revision of the Nature Conservation Act, which had been underway since 1960 (Rassi et al., 1986). Full revision of the law entered into force in 1997, which led to a reduction in law‐related proposals and, consequently, partly explained the absence of trends in deep leverage impacts in the quantitative analysis. It is noteworthy that there was also a change in operating culture, as later on, the mandate of these working groups did not include proposing explicit changes to laws (E. Hyvärinen, personal communication, 2024).

Another likely driver explaining some of our results is the decreasing funding of environmental administration. The funding of the Ministry of Environment (excluding housing expenditure) decreased over the study period from ∼1% of the national budget to around 0.3–0.5% in the 2000s (Ministry of Finance, 2024). This austerity probably contributed to fewer proposals for costly conservation programs and the overarching emphasis on cost‐effectiveness, engaging the private sector, as well as to the continuous appeals for ensuring funding for conservation, research, and the RL assessments themselves, perpetuating an undertone that biodiversity research and conservation measures must constantly justify their existence and resource use in society.

A noteworthy trend was the shift from top‐down conservation to voluntary measures. The implementation of the EU's Natura 2000 program caused severe resistance among stakeholders in Finland and contributed to the development of voluntary policies (Hiedanpää, 2005), visible also in our results. The development of voluntariness reflects the principles of just transformation, with increased inclusivity, legitimacy, and shifts in power relations (Fougères et al., 2022; Lempinen & Vainio, 2023; Massarella et al., 2021; Paloniemi & Vainio, 2011). Numerous proposals gave advice on the implementation of the Forest Biodiversity Program of Southern Finland (METSO), which since 2008 has played a key role in mitigating tensions in the forest conservation arena, bringing together all the major stakeholders from nature conservation, forest biodiversity science, administration, and forestry.

The numerous action proposals related to biological research may reflect the assessors’ and steering group members’ educational backgrounds, which are typically in natural, rather than social, sciences. Hence, knowledge production may be seen as a way to shift paradigms and engage deeper leverage points (1–3) through improved awareness. An obvious reason for the enduring prevalence of land and water management actions (1.1–1.2) and conservation area designation and planning (6.1–6.4) is that land‐use change remains the most important direct driver of biodiversity loss (IPBES, 2019). In the short term, these actions are an ecological precondition for the survival of threatened species and ecosystems. This has received much attention in the international policy arena in the form of different area‐based targets (Gurney et al., 2023). However, the composition of the steering group responsible for the general proposals was more diverse for the action plan of 2017. It had representatives of several ministries, forest industries, conservation nongovernmental organizations, municipalities, and associations of farmers and forest owners. This probably contributed to the higher spread among action categories and most cross‐sectoral proposals among species assessments, reflecting the increasing societal aspirations for mainstreaming. Even though cross‐sectorality was increasing prior to the last assessment, higher inclusivity in drafting the proposals should increase their legitimacy and actionability (Lemos et al., 2018; Matuk et al., 2020; Moser, 2016; Visseren‐Hamakers et al., 2021). One long‐term RLE assessor reported in an interview that their employer had halted efforts to include recommendations explicitly concerning transformative change because these were not commissioned from the assessment (Salomaa & Arponen, 2023).

### The role of RLs in transforming society

There is an ongoing discussion on the demand for policy‐prescriptive science and increasing calls for scientists to actively participate in transforming society. Beck et al. (2022) suggest that, for assessments (e.g., by IPBES) to be solution‐oriented and advance transformative change, they and the institutions performing them must transform themselves. The IUCN has been recognized as a boundary organization (Lidskog, 2014) that leads the adoption of policies, analyses, and norms of the worldwide conservation community (Stuart et al., 2017). However, RLs are rather different in character from the global assessments referred to by Beck et al. (2022). They have a narrower scope and specific scientific criteria, and their detachment from policy has been emphasized as a requirement for their strategic significance (Lidskog, 2014). Clearly, the threat assessment process should rely on ecological expertise and remain independent. Nonetheless, engaging actors across society even more broadly and, importantly, including scientific expertise, especially in policy research and societal change in the process of devising the action proposals, could improve their actionability. It is also critical to acknowledge that biodiversity loss takes place locally and gradually; transformative change is also needed in local‐scale policies to ensure timely, preventive actions (Small Wins pathway) (Termeer et al., 2024).

The diverse impacts of RLs are noted in the literature. RLs of ecosystems are used to guide national legislation, land‐use planning, land management, monitoring, and reporting in many countries (Bland et al., 2019; Botts et al., 2020). The U.S. Endangered Species Act contains a similar approach to listing species according to their extinction risk, which has influenced legislation and policies in many countries (Gronewold, 2023). Researchers have identified a plethora of potential uses for RLs, ranging from natural capital accounting to informing business decisions (Alaniz et al., 2019; Bland et al., 2019). Nicholson et al. (2024) found that RLs can have important roles in 16 of the 23 targets that contribute to the implementation of the Global Biodiversity Framework, covering many threat reduction, meeting needs, and mainstreaming targets. RLs guide conservation funding (Betts et al., 2020) and contribute to the perception of biodiversity loss as a global crisis (Betts et al., 2020; Lidskog, 2014). Although none of these impacts have been explicitly assessed from the perspective of transformative change, they align with our findings at the level of conservation actions.

A central tenet of transformative change is a profound paradigm shift in society (IPBES, 2024; Meadows, 1999; Raymond et al., 2021; West et al., 2020). Rather than merely transmitting scientific knowledge to the society, RLs are cocreated between scientists and the society (Jørstad & Skogen, 2010; Salomaa & Arponen, 2023). The trends we observed here similarly reflect broader changes in society and paradigms of conservation (Mace, 2014). The failure to halt biodiversity loss despite positive developments may stem from the marginal position of a holistic understanding of humans as part of nature, often described as a potential way out of the current unsustainable development pathway (Chan et al., 2016; Mattijssen et al., 2020; Raymond et al., 2021; West et al., 2020). Indeed, not even the more progressive action proposals (e.g., novel economic instruments) reach the deepest leverage points. Instead, they align with a dualistic thinking of nature and society, appealing to neoliberal institutions and actors that are inherently resistant to transformative change (West et al., 2020), thereby possibly even inhibiting a paradigm shift. This is also supported by our observation that nonmonetary values that could promote a shift from economy‐driven thinking were not proposed as incentives, apart from a single mention in 2019. Even though some actions capable of producing paradigm‐level effects were present, they may not have the desired impact unless they are harnessed for a shift specifically toward a more sustainable paradigm. In addition, a simple delay between conception and wide‐scale adoption of new ideas may manifest as ineffectiveness of proposals, lacking the quick dimension in Termeer et al.’s (2024) framework.

Even though we assessed only action proposals and not their fulfillment, our results indirectly reflect the realized development because proposals were repeated only when they had not been (adequately) addressed or were considered to have become unnecessary or unrealistic. Many action proposals have been put into practice (Hyvärinen et al., 2019; Juslén & Sirkiä, 2013; Kontula & Raunio, 2018; Rassi et al., 2010). It appears that the RL has had a role in the development and mainstreaming of biodiversity conservation in Finland, which is also supported by the perceptions of RL assessors (Salomaa & Arponen, 2023). However, some specific issues recognized in the assessments nearly four decades ago are only now starting to get the necessary attention or are still not being addressed. Moreover, some actions reaching deep leverage were almost or totally absent from our data. For example, nonmonetary incentives, systemic litigation, and consideration of the rights of nature under legal frameworks could provide opportunities for deeper leverage of action proposals or other strategies and recommendations developed based on them.

RLs do have a role in transformative change, but because they were not designed to promote it, other complementary tools and processes are needed where transformative change is an explicit goal. For example, the development of the National Biodiversity Strategy and Action Plan should account for it, and there have been efforts to promote transformative thinking in that context in Finland (Hyysalo et al., 2019). The IPBES transformative change assessment is also expected to make a global contribution toward advancing transformative policies and thinking (IPBES, 2024).

### Limitations of our approach

The first cross‐sectoral action proposals in the RLs emerged before biodiversity mainstreaming became a political priority (e.g., CBD, 2018) and may be more coincidental than intentional promotion of transformative change. We considered the importance of cross‐sectorality to stand regardless of the underlying motives, but our simple assessment may not accurately reflect the development of transformative ambitions. Similarly, the overall shift to actions with higher transformative potential may simply be a consequence of observing that prior actions have been insufficient (Hilton et al., 2023), rather than a strategic shift to transformative principles. Nonetheless, we do not see this as a major limitation because the concept of transformative change itself was conceived as a response to the ineffectiveness of prior action (IPBES, 2019).

It is also possible that the increased complexity of proposals is partly a consequence of wanting to reduce the number of action proposals to shorten the book sections, but this is unlikely to explain the entire trend, as it coincides with increasingly cross‐sectoral proposals. We also acknowledge a trade‐off between perfectly comprehensive and highly ambitious versus actionable proposals. Although complexity can ideally mean that the implementation of an action is improved by integrating it with others (e.g., ensuring the acceptance and correct implementation of an action through outreach and training), it is also possible that it can lead to overly complicated situations and reduced feasibility. Assessing the feasibility of increasingly complex actions goes beyond the scope of our study.

Our analyses were limited to a single case study country, and many of the individual findings were clearly case specific. Finland is a relatively distinctive case with a long history of extensive RL assessments, although long series of assessments are also found, for example, in Norway, South Africa, and Sweden (NBIC, 2021; SANBI, 2024; SLU Artdatabanken, 2020). Nonetheless, we think that the Finnish dataset provides a unique occasion for a comprehensive historical perspective that is not easily achievable in other contexts. We also believe that the general findings hold beyond country borders because they are supported by previous research on the role of RLs in the science–policy interface.

Concerning our analytical framework, some action categories are highly heterogeneous regarding their leverage (Arponen & Salomaa, 2023), and for future studies, the approach would benefit from a further level of subcategories. This would be most critical for different outreach approaches and types of legislation that have a wide spread in their leverage points. This issue, together with the fact that most action categories are associated with multiple leverage points, makes the approach rather coarse and could be a partial reason why the trends in leverage points were weak in our quantitative analyses.

We observed a gradual development in RL action proposals toward deeper leverage and cross‐sectorality over four decades. This trend is probably both a driver and a consequence of the same trend in society as a whole, driven by the ongoing loss of biodiversity. Despite a positive direction, the transformative potential has not materialized to an extent that could turn the tide for biodiversity. Cross‐sectorality has increased but often without reaching the deepest leverage points and at an inadequate pace; hence, it has not led to truly transformative changes.

We conclude that RLs play a role in transformative changes, but it can be further amplified. Although we focused on one country, where the devising of action proposals has already taken a step toward higher inclusiveness, we emphasize that, regardless of location, complementary processes are required to facilitate the broader adoption of such proposals and to enable transformative change. The value of our analytical approach extends beyond the context of RL assessments to analyzing, for example, conservation projects, policies, and strategies. Our results also accentuate the importance of considering complementary dimensions of transformative change simultaneously to achieve a comprehensive understanding of viable paths to societal change.

## Supporting information



Supporting information

Supporting information

Supporting information

Supporting information

Supporting information

Supporting information
