# Supplementary material for: Changes in the transformative potential of action proposals in Finnish Red Lists from 1986 to 2019
Source: Conserv Biol. 2026 May 6;40(4):e70312. doi: 10.1111/cobi.70312 (PMC13392750; doi:10.1111/cobi.70312)
Supplement: Supplementary file 1 — Supporting information [file COBI-40-e70312-s001.pdf]

Appendix S1: Details on Red List assessment work groups and the process. Guidelines for coding Red List quotations to conservation action categories.

Red List assessment process in Finland

Finland has continuously working taxonomic and habitat type expert groups, composed of researchers, environmental administration experts and professional-level enthusiasts. Each group is supervised by its own steering group appointed and led by the Ministry of the Environment. The main duty of the expert groups is to carry out threat assessments at circa ten-year intervals and maintain and improve the knowledge base required for assessments. They also provide important expert assistance to the authorities on issues related to species and habitat conservation. All the assessments are done by expert groups and approved by steering groups. The steering groups also write the general action proposal sections in the assessments, while the expert groups write the proposals concerning specific species and habitats. The steering groups include members from Finnish environmental and natural resources administration and research organizations, and in the case of the 2017 Action plan, also representatives of stakeholder groups.

Guidelines for coding

Coding the data required drawing partly subjective lines between ambiguous action categories and interpreting ambiguous recommendations. Here we describe such choices and our reasoning behind them. In the taxon-specific text sections we coded only the text that was explicitly formulated as action proposals, in order to have a clearly delimited sample. As these sections were written by different expert groups, there was some variation for example in how much they focused on identifying problems or goals (that were not coded) vs. proposing solutions (that were coded), but this did not present a systematic bias regarding the overall trend, as the variation was within each assessment.

Distinction between 1.1. Site/area stewardship and 2.1. Species stewardship was often difficult for recommendations regarding species' habitats. When the recommendations targeted specific resources for the species, such as ensuring the persistence of a food plant, nesting sites, or protecting the species' host trees, we considered it as 2.1. Species stewardship. Sometimes similar recommendations were described as more general site management actions, but urging to include considering the habitat requirements of the species, we interpreted them as both 1.1. site/area stewardship and 2.1. Species stewardship. For example, "in site management one must ensure deadwood continuum of aspen", implies broader site management actions, but with special attention to the species' critical resources.

As described in the original Action classification categories, the distinction between 1.1 Site/area stewardship vs. 1.2. Ecosystem & natural process (re)creation is partly a matter of scale. In our data, especially recommendations regarding semi-natural grasslands and their management through mowing or grazing were often ambiguous, but we sought to classify actions on local/specific sites as 1.1. Site/area stewardship, while more general recommendations to "maintain grazing" without specific locations were assigned under 1.2 Ecosystem & natural process (re)creation.

"Securing genetic purity" without specific reference to captive breeding and reintroduction actions was coded under 2.1. Species stewardship, with the assumption that it would entail general population management actions only.

Proposals to enhance fisheries control overall without a specific target were classified under 4.1. Detection & arrest only, but when the proposal discussed, for example, improving the surveillance of raptor nests, it was coded additionally under 2.1 Species stewardship, or proposals to improve oversight of compliance of the laws that protect spring habitats were additionally coded under 1.1. Site/Area Stewardship. "Enhancing the maintenance of habitats protected by the Forest Act" was quite ambiguous, but we coded this as 4.1. Detection & arrest, assuming the proposal was addressing challenges in compliance.

In 5.2. Better products & management practices we included only recommendations that contained some novel idea, method or policy (e.g. an online platform for offering and requesting pastures for rent, constructed wetlands, different kinds of experimental pilot projects), or a broader change in practices (such as the concept of planning for multifunctionality in forestry), that would have a role as an incentive, while simple instructions and restrictions, such as "avoid cutting down common hazels in commercial forest management", went under 7.2. Policies & guidelines.

As mentioned in the Action classification description, the category 6.1 Protected Area Designation &/or Acquisition could have equally well been described as a subsection of 7.1. Laws, regulations & codes, and by default it contains changes in legislation required for PA designation, therefore, proposals regarding protected areas were coded under 6.1. only and not 7.1., unless they were discussed in the context of some other (broader) change to legislation.

There were only a few proposals that could perhaps have been considered as 6.2. Easements & resource rights, but as they are quite ambiguous in the Finnish legal context, and simultaneously coded under other action categories, we omitted this category altogether.

Proposals that emphasized the importance of targeting conservation actions to most important locations were interpreted as 6.4. Conservation planning even though any planning process or method was not necessarily implicitly mentioned.

Proposals to fence out visitors, re-route trails and such to protect species or sensitive habitats were considered under 1.1. Site/Area Stewardship (the category contains "Visitor management"), while site infrastructure development in the sense of capital investment was absent from the proposals (6.5. Site

infrastructure). Needs regarding infrastructure may not be seen as critical for conservation in Finland where protected areas are not fenced and e.g. poaching is not focused on PAs in particular, and basic infrastructure to prevent littering, to keep visitors on trails and designated campfire sites tends to be well organized by default.

There were very fine differences regarding how collecting butterflies was addressed, but we interpreted the recommendations literally based on their strictness: “collecting the butterflies must be avoided” was considered under 7.2. Policies & guidelines, while “collecting the butterflies must not be allowed” went under 7.1 Laws, regulations & codes.

One contribution of the RL assessments has been to propose lists of species for the status of “species under strict protection” in the Nature Conservation Decree. The introduction of this status was first proposed in the section of the 1986 species assessment regarding the revision of the Nature Conservation Act from 1923, where we coded it as a novel law proposal (7.1 Laws, regulations & codes). Later on, the action proposals often suggested assigning this status to specific species. However, the implications of this status have changed over the years. The status requires that important occurrences of the species must not be deteriorated or destroyed, and it allows for strict protection of sites inhabited by the species, for which we coded all of these (species-specific) proposals under 6.1. Protected Area Designation &/or Acquisition. Originally it was also proposed that a conservation program would be developed for each of the listed species, but this changed in the RL assessment of 2010 because it had turned out to be infeasible – only 25 of the >600 listed species had had their programs developed during the preceding 10 year-period – and it was recommended that programs would be from then on developed for “the most endangered species only, the conservation of which involves several parties and complicated issues, and for which the programs are therefore necessary”. Based on this information, we coded the proposals for assigning the status to a species prior to 2010 also as 6.4. Conservation Planning regardless of whether planning or a program was directly mentioned, but excluded this code starting from 2010 unless planning was explicitly mentioned.

Proposals for research on feasibility of or possibilities for translocation were classified under 8.1 Basic Research & Status Monitoring unless testing was specifically mentioned, while testing it qualified as 8.2 Evaluation, Effectiveness Measures & Learning.

The RLE assessments contained some proposals that seemed more like goals and targets rather than concrete actions, such as improving the water quality in running waters without any indication of how to achieve the improvement. Such proposals were coded only as general proposals, possibly cross-sectoral, but not associated with action categories, and therefore these are absent from the majority of our results.

Removal of dams/obstructions and construction of fish ladders were considered as cross-sectoral, as they are mainly linked with energy production rather than fisheries regulation (and because a large part of Finnish rivers and streams are still today obstructed).

Proposals to develop advising for landowners and farmers regarding different options to engage in conservation actions or reduce negative impact of their (professional) activities were coded both as 3.1.

Outreach & communication and as 9.2. Training/capacity development, because these activities increase their overall awareness of such issues but also improve their professional capacity to engage in such actions.

Many proposals addressed openness of data and increase of knowledge transfer. Proposals that dealt with e.g. establishing new databases we classified under 10.1. Internal organizational management & administration (the description of which contains ”provision of tech & facilities”), while transfer of knowledge across organizations or stakeholder groups we interpreted as 10.3. Alliance & partnership development.
