# Supplementary material for: Changes in the transformative potential of action proposals in Finnish Red Lists from 1986 to 2019
Source: Conserv Biol. 2026 May 6;40(4):e70312. doi: 10.1111/cobi.70312 (PMC13392750; doi:10.1111/cobi.70312)
Supplement: Supplementary file 2 — Supporting information [file COBI-40-e70312-s005.pdf]

Appendix S2: Association of conservation actions and leverage points

Association of conservation actions and leverage points used in this study. Adapted from Arponen & Salomaa (2023), sustainability leverage points by Meadows (1999). The circles describe the presence of an association between conservation actions and leverage points. Here we have removed associations that were not relevant in our dataset, as well as action categories that were not present in our data. From action 4.2 Criminal Prosecution & Conviction we removed leverage point 5 Rules that concerned courts establishing new rules through e.g. precedents, which were absent from our data. Under 4.3 Non-Criminal Legal Action class actions and systemic litigation (e.g., climate change litigation) were not mentioned and hence we removed leverage point 4 System Structure and leverage point 3 Goals from this category. Under 7.1 Laws, Regulations and Codes leverage point 2 Paradigms and leverage point 3 Goals were linked only with law proposals regarding rights of nature, which were absent and thus removed.

LEVERAGE POINTS	LP12 Parameters	LP11 Buffers	LP10 Stock structure	LP9 Delays	LP8 Control loops	LP7 Driving loops	LP6 Information	LP5 Rules	LP4 System structure	LP3 Goals	LP2 Paradigms	LP1 Transcendence
A. TARGET RESTORATION / STRESS REDUCTION ACTIONS												
1. Land / Water Management												
1.1 Site/Area stewardship	●	●	●									
1.2 Ecosystem & Natural Process (Re)Creation	●	●	●	●	●	●						
2. Species Management												
2.1 Species Stewardship	●	●	●	●								

	2.2 Species Re-Introduction & Translocation	●	●	●	●		●						
	2.3 Ex-Situ Conservation	●	●	●	●								
B. BEHAVORIAL CHANGE / THREAT REDUCTION ACTIONS													
3. Awareness Raising													
	3.1 Outreach & Communications	●	●	●	●	●	●	●	●	●	●	●	●
4. Law Enforcement & Prosecution													
	4.1 Detection & Arrest	●	●	●		●	●	●					
	4.2 Criminal Prosecution & Conviction					●	●	●					
	4.3 Non-Criminal Legal Action					●		●	●				
5. Livelihood, Economic & Moral Incentives													

	5.1 Linked Enterprises & Alternative Livelihoods	●	●	●		●	●			●			
	5.2 Better Products & Management Practices	●	●	●									
	5.3 Market-Based Incentives	●	●	●		●	●	●	●				
	5.4 Direct Economic Incentives	●	●	●		●	●		●				
	5.5 Non-Monetary Values	●	●	●							●	●	
C. ENABLING CONDITION ACTIONS													
6. Conservation Designation & Planning													
	6.1 Protected Area Designation &/or Acquisition	●	●	●	●	●	●		●				
	6.3 Land/Water Use Zoning & Designation	●	●	●	●	●	●		●				
	6.4 Conservation Planning							●					

7. Legal & Policy Frameworks												
7.1 Laws, Regulations & Codes	●	●	●		●		●	●	●			
7.2 Policies & Guidelines	●	●	●		●		●	●	●			
8. Research & Monitoring												
8.1 Basic Research & Status Monitoring					●		●					
8.2 Evaluation, Effectiveness Measures & Learning					●		●					
9. Education & Training												
9.1 Formal Education							●		●		●	●
9.2 Training & Individual Capacity Development							●		●		●	●
10. Institutional Development												
10.1 Internal Organizational Management & Administration	●	●	●		●	●						

	10.2 External Organizational Development & Support					●		●		●			
	10.3 Alliance & Partnership Development							●		●	●	●	
	10.4 Financing Conservation	●	●	●		●	●						
