# Supplementary material for: Changes in the transformative potential of action proposals in Finnish Red Lists from 1986 to 2019
Source: Conserv Biol. 2026 May 6;40(4):e70312. doi: 10.1111/cobi.70312 (PMC13392750; doi:10.1111/cobi.70312)
Supplement: Supplementary file 3 — Supporting information [file COBI-40-e70312-s004.pdf]

Appendices S3-S14: Quantitative analysis of Red List action proposal codes and leverage categories

Appendix S3. Supplementary methods and results

Methods

Distribution of action proposal codes and the associated Leverage Points across different types of action proposals

We tested whether action proposal codes were differently distributed into action categories across the three types of action proposals (general or species-specific action proposals of species assessments or action proposals of ecosystem assessments), to determine whether different types of assessments can be joined for testing for temporal trends. For this purpose, we pooled action proposal codes of each of the three types together across years, and used Pearson's X^2 test of independence to test for differences in their distributions in pairwise comparisons across the three types. To avoid small frequencies of action proposal codes per category, we grouped the action proposals into main-level action categories for the analysis. Similarly, we tested whether leverage categories (Meadows 1999, Arponen & Salomaa 2023) were differently represented across the three types of action proposals they were associated with, but without pooling leverage categories into larger groups. All quantitative analyses were performed using R Statistical Software (v4.4.1; R Core Team 2024).

Temporal trends in the distribution of action proposal codes and associated Leverage Points

We tested for temporal trends in the proportional shares of action proposal codes over time using a non-parametric Mann-Kendall trend test (Sen 1968, Newson 2002, Chen 2022). Mann-Kendall trend tests were performed using the Kendall R package (v2.2.1; McLeod 2022). As action proposal codes differed from each other in a statistically significant manner across all types of action proposals, we carried out the test separately for general and species-specific action proposals of species assessments. Temporal trends in action proposals of ecosystem assessments were not tested due to the lack of enough time points in the data. Similarly, we tested for temporal trends in the distribution of leverage categories over time. Since there were no statistically significant differences in the distribution of leverage categories associated with general action proposal codes of species-specific and ecosystem assessments, we pooled these publications together for the analysis. We carried out the analysis of temporal trends otherwise in a similar manner as in the case of action proposal codes.

A non-parametric test was selected for testing temporal trends in the proportional shares due to a large number of zeros in the data, which limited the possibility of using beta or logistic regression for testing a linear trend. We first studied the statistical significance and direction of the trend (the value of Kendall's τ correlation coefficient) of individual action proposal codes or leverage categories. Next, we sorted the categories as per their Kendall's τ correlation coefficient and calculated the cumulative

proportion of action proposal codes or leverage categories that had shown a non-increasing temporal trend as per their Kendall's τ correlation over time. This measure was used as a proxy for the scale of overall temporal change in the proportional shares and composition of the distributions. Finally, we used a linear model to test for the strength and linearity of this overall temporal change. A linear model was used despite proportional data, as the cumulative proportions ranged in mid-range values rather than close to 0 or 1.

Temporal trends in cross-sectorality and complexity of action proposal codes

We studied the temporal development of the complexity and cross-sectorality of action proposal codes by using linear models, where either complexity or cross-sectorality was the response variable, and the publication year and the type of action proposal (general or species-specific action proposals of species assessments or action proposals of ecosystem assessments) were explanatory variables. Complexity was measured by number of action proposal codes per the number of total quotations and cross-sectorality was measured by the number of quotations marked as cross-sectoral per the number of total quotations. For more details on the measurement of cross-sectorality and complexity, see the main text. We also studied the proportional shares of cross-sectoral action proposal codes (i.e. ones that were derived from quotations marked as cross-sectoral) out of all action proposal codes across the three different types of action proposals and years of publication.

Results

Number and distribution of different types of action proposal codes

Action category codes associated with the proposals were unevenly distributed across publications, with the 2019 species assessment containing 60 % of all action codes in general action proposals of species assessments, and the 1986 species assessment containing 83 % of all action codes associated with species-specific action proposals.

The distribution of species-specific action proposal codes into main action categories differed from general (X^2 = 483.15, df = 9) and from ecosystem-specific action proposals (X^2 = 374.41, df = 9) more than the latter two categories differed from each other (X^2 = 67.199, df = 9), but the differences in distribution were statistically significant across all three pairwise comparisons (*p*-value < 0.01; Appendix S4 panel a).

The distribution of leverage categories associated with species-specific action proposal codes differed from leverage categories associated with general action proposal codes of species-specific (X^2 = 287.77, df = 11) and ecosystem (X^2 = 160.57, df = 11; *p*-values < 0.01) assessments, but the latter two categories did not differ in a statistically significant manner from each other (X^2 = 10.445, df = 11, *p*-value = 0.4909; Appendix S4 panel b).

Temporal trends in the distribution of action proposal codes into action categories

When action proposal codes were sorted by their increasing or decreasing tendency in terms of their proportional shares among action categories (Fig. 3, main text), the cumulative share of the 14 non-increasing general action proposal categories decreased from 70.7 % to 35.1 % over the years (*p*-value < 0.001 ***, $F_{1,3}$ = 344.1, R^2 = 0.9914). Similarly, the cumulative share of the 10 non-increasing species-specific action proposal categories decreased from 56.1 % to 15.4 % (*p*-value > 0.05 *, $F_{1,2}$ =

83.2, R^2 = 0.9765). However, individual action points did not demonstrate statistically significant increasing or decreasing temporal trends in their proportional shares over the study period (Appendices S5-S7).

Temporal trends in the distribution of leverage categories across years

When leverage categories were sorted by their increasing or decreasing tendency in terms of their proportional shares, the cumulative share of the 7 non-increasing leverage categories associated with general action proposals of species and ecosystem assessments decreased from 72.5 % to 65.4 % over the years (*p*-value < 0.001 ***, $F_{1,3}$ = 55.09, R^2 = 0.9168, Fig. 3 main text, Appendix S8), indicating a statistically significant but small change over time. The cumulative share of the 5 non-increasing leverage categories associated with species-specific action proposals also decreased from 59.7 % to 46.8 % over the years, but the trend was not statistically significant (*p*-value > 0.05, $F_{1,2}$ = 10.32, R^2 = 0.8376, Appendix S9). LP5 demonstrated a very small increase among leverage categories associated with general action proposal codes of species-specific and ecosystem assessments (Appendices S5 and S8).

Temporal trends in cross-sectorality and complexity of action proposals

Complexity and cross-sectorality of action proposals increased over time, general action proposals being most complex and ecosystem-specific action proposals being most cross-sectoral (Fig. 4, main text, Appendices S10-S11). Proportional shares of cross-sectoral out of all action proposal codes varied differently across action categories in the three types of action proposals, being largest for the action codes of ecosystem assessments (Appendices S12-S14).

References

Arponen, A., Salomaa, A., 2023. Transformative potential of conservation actions. Biodivers. Conserv. 32: 3509-3531. https://doi.org/10.1007/s10531-023-02600-3

Chen, S. (2022). Practical guide to using Kendall's τ in the context of forecasting critical transitions. *R Soc Open Sci.* 9: 211346. DOI: 10.1098/rsos.211346.

McLeod A (2022). _Kendall: Kendall Rank Correlation and Mann-Kendall Trend Test_. R package version 2.2.1, <https://CRAN.R-project.org/package=Kendall>.

Meadows, D., 1999. Leverage Points — Places to Intervene in a System, The Sustainability Institute.

Newson, R. (2002). Parameters behind "nonparametric" statistics: Kendall's tau, Somers' D and median differences. *The Stata Journal* 2: 45-64. DOI: 10.1177/1536867X0200200103.

R Core Team (2024). _R: A Language and Environment for Statistical Computing_. R Foundation for Statistical Computing, Vienna, Austria. <https://www.R-project.org/>.

Sen, P. K. (1968). Estimates of the regression coefficient based on Kendall's Tau. *Journal of the American Statistical Association* 63: 1379-1389. DOI: 10.1080/01621459.1968.10480934

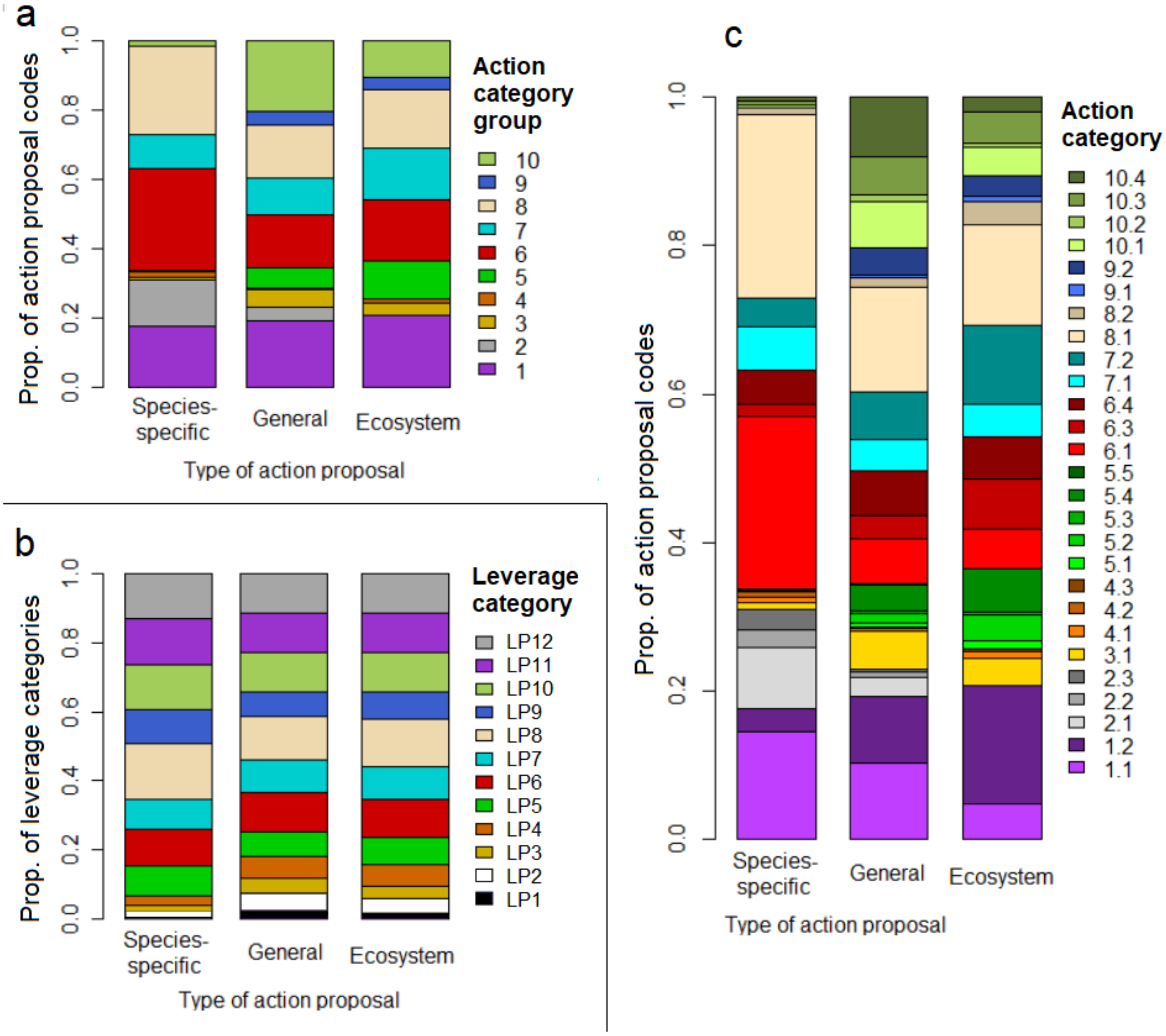

Appendix S4: Pooled (a) and detailed (c) action categories (colours) of the three types of action proposals (x axes), displayed as per their proportional shares of all action proposal codes in the category (y axis). b: Representation of leverage categories (colours) across the three types of action proposals they are associated with (x axis) as per their proportional shares of all leverage category associations in the category (y axis).

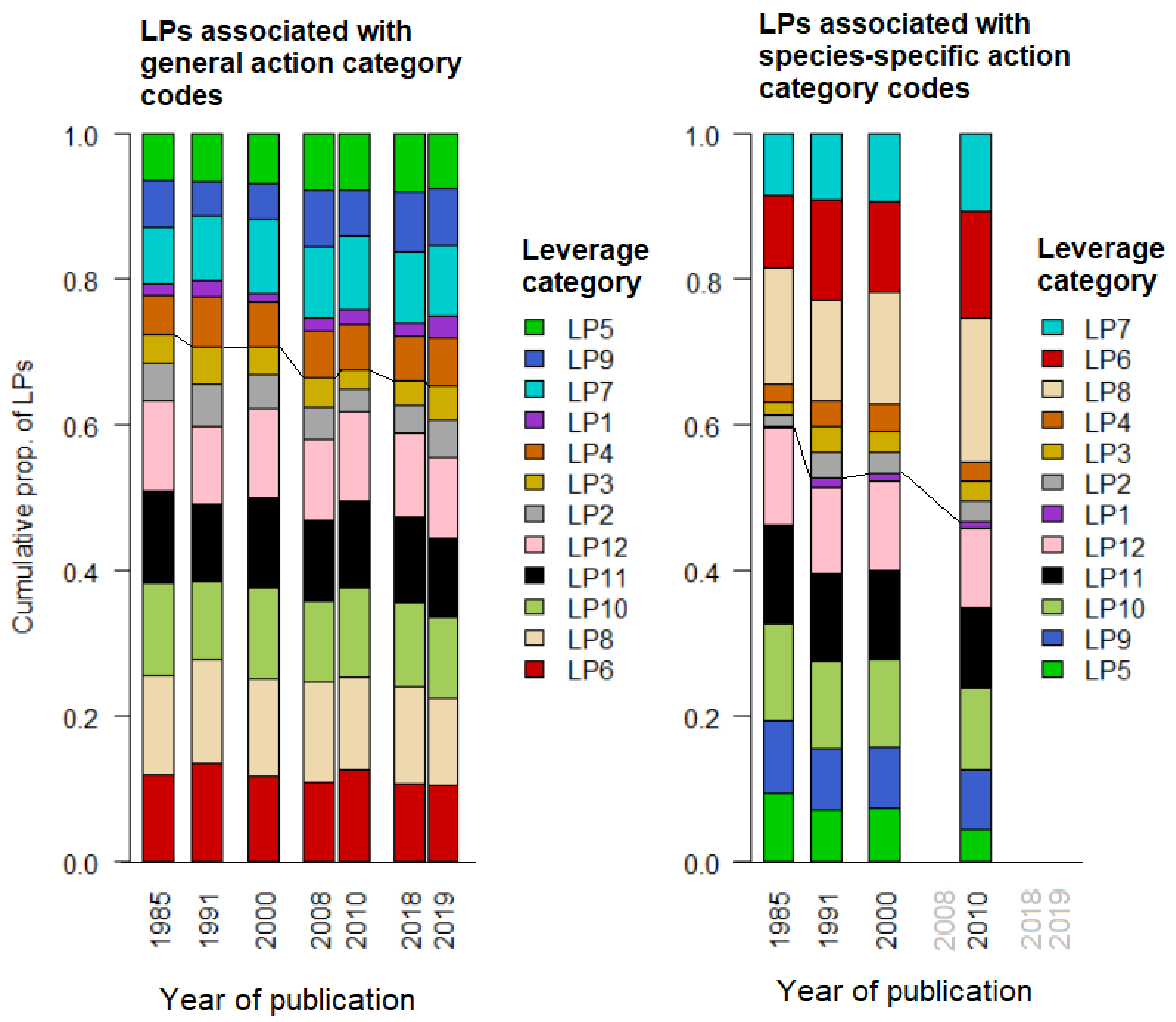

Appendix S5: Proportional shares (y axis) of leverage categories associated with general species-specific action proposals (panel a) categories and the associated leverage categories (panel b) of species and ecosystem assessments, sorted by the time of publication (x axis) and the value of Kendall's *τ* of the action category, from the most negative (bottom) to the most positive (top). For readability, results are shown only for action categories with >= 5 % share for at least one year.

Appendix S6: Temporal trends in the proportional shares of general action proposal codes of species assessments, as indicated by the statistical significance (*p* value) of Kendall's rank correlation coefficient *τ* and by the variance of Kendall Score (var(S)) of Mann-Kendall's non-parametric test of temporal trend. The results have been sorted by the value of *τ*. Action categories that resulted in Kendall's *τ* |>0.5| shown with blue shading. For details and further references of Kendall's rank correlation coefficient and the Kendall Score, see McLeod (2022).

**Action category**	*τ*	***p* value**	**var(S)**
5.5	0.6325	0.2888	8.00000
5.3	0.6325	0.28885	8.00000
5.2	0.6325	0.28885	8.00000
5.1	0.6325	0.2888	8.00000
10.3	0.6000	0.2207	16.67
7.2	0.6000	0.2207	16.67
1.2	0.6000	0.2207	16.67
6.4	0.4000	0.4624	16.67
3.1	0.4000	0.4624	16.67
1.1	0.4000	0.4624	16.67
10.1	0.2000	0.8065	16.67
5.4	0.2000	0.8065	16.67
4.1	0.1195	1.0000	13.00
10.4	0.0000	1.0000	16.67
9.2	0.0000	1.0000	16.67
6.3	0.0000	1.0000	16.67
10.2	-0.1054	1.0000	15.67
4.3	-0.1195	1.0000	13.00
2.3	-0.1195	1.0000	13.00
2.2	-0.1195	1.0000	13.00
6.1	-0.2000	0.8065	16.67
9.1	-0.3162	0.6134	15.67
8.2	-0.3162	0.6134	15.67
2.1	-0.3162	0.6134	15.67
4.2	-0.6325	0.2888	8.00
8.1	-0.8000	0.0864	16.67
7.1	-0.8000	0.0864	16.67

Appendix S7: Temporal trends in the proportional shares of species-specific action proposal codes, as indicated by the statistical significance and sorted by Kendall's *τ* of Mann-Kendall's non-parametric test of temporal trend. Presented parameters and sorting as in Table C1

**Action category**	*τ*	***p* value**	**Var(S)**
1.2	1.0000	0.0894	8.667
10.3	0.6667	0.3082	8.667
10.1	0.6667	0.3082	8.667
8.2	0.6667	0.3082	8.667
8.1	0.6667	0.3082	8.667
6.3	0.6667	0.3082	8.667
1.1	0.6667	0.3082	8.667
3.1	0.3333	0.7341	8.667
5.4	0.1826	1.0000	7.667
4.1	0.1826	1.0000	7.667
6.4	0.0000	1.0000	8.667
10.4	-0.3333	0.7341	8.667
7.2	-0.3333	0.7341	8.667
7.1	-0.6667	0.3082	8.667
6.1	-0.6667	0.3082	8.667
2.3	-0.6667	0.3082	8.667
2.2	-0.6667	0.3082	8.667
5.2	-0.7071	0.3711	5.000
4.2	-0.7071	0.3711	5.000
2.1	-0.9129	0.1486	7.667

Appendix S8: Temporal trends in the proportional shares of leverage categories associated with general action proposals of species and ecosystem assessments, as indicated by the statistical significance and Kendall's tau of Mann-Kendall's non-parametric test of temporal trend. Presented parameters and sorting as in Table C1

	τ	***p* value**	**Var(S)**
LP5	0.7143	0.03550 *	44.33
LP9	0.5238	0.1331	44.33
LP7	0.5238	0.1331	44.33
LP1	0.4286	0.2296	44.33
LP4	0.2381	0.5480	44.33
LP3	-0.2381	0.5480	44.33
LP2	-0.2381	0.5480	44.33
LP12	-0.3333	0.3675	44.33
LP11	-0.3333	0.3675	44.33
LP10	-0.3333	0.3675	44.33
LP8	-0.5238	0.1331	44.33
LP6	-0.6190	0.0715	44.33

Appendix S9: Temporal trends in the proportional shares of leverage categories associated with species-specific action proposals of species and ecosystem assessments, as indicated by the statistical significance and Kendall's tau of Mann-Kendall's non-parametric test of temporal trend. Presented parameters and sorting as in Table C1

	τ	***p* value**	**Var(S)**
LP7	1.0000	0.0894 .	8.667
LP6	0.6667	0.3082	8.667
LP4	0.3333	0.7341	8.667
LP8	0.3333	0.7341	8.667
LP1	0.0000	1.0000	8.667
LP2	0.0000	1.0000	8.667
LP3	0.0000	1.0000	8.667
LP5	-0.6667	0.3082	8.667
LP9	-0.6667	0.3082	8.667
LP10	-0.6667	0.3082	8.667
LP11	-0.6667	0.3082	8.667
LP12	-0.6667	0.3082	8.667

Appendix S10: Parameters of a linear model ($R^2 = 0.837$, $F_{3,7} = 12.0$, p-value < 0.01 **) of the complexity of action proposals as a function of publication year and the type of action proposal. Asterisks (*) indicate statistical significance (p-value < 0.05).

	Estimate	Std. error	*T* value	Pr(>|t|)	
(Intercept)	-41.6	13.5	-3.07	0.0180	*
Publication year	0.022	0.00672	3.23	0.0146	*
General action proposals of species assessments	0.562	0.209	2.70	0.0308	*
Species-specific action proposals of species assessments	-0.120	0.228	-0.525	0.616	

Appendix S11: Parameters of a linear model ($R^2 = 0.934$, $F_{3,7} = 33.1$, p-value < 0.001 ***) of the cross-sectorality of action proposals as a function of publication year and the type of action proposal. Asterisks (** or *) indicate statistical significance (p-value < 0.01 or < 0.05, respectively).

	Estimate	Std. error	*T* value	Pr(>|t|)	
(Intercept)	-15.9	3.50	-4.55	0.00265	**
Publication year	0.00818	0.00174	4.71	0.00219	**
General action proposals of species assessments	-0.140	0.0539	-2.59	0.03590	*
Species-specific action proposals of species assessments	-0.309	0.0589	-5.25	0.00119	**

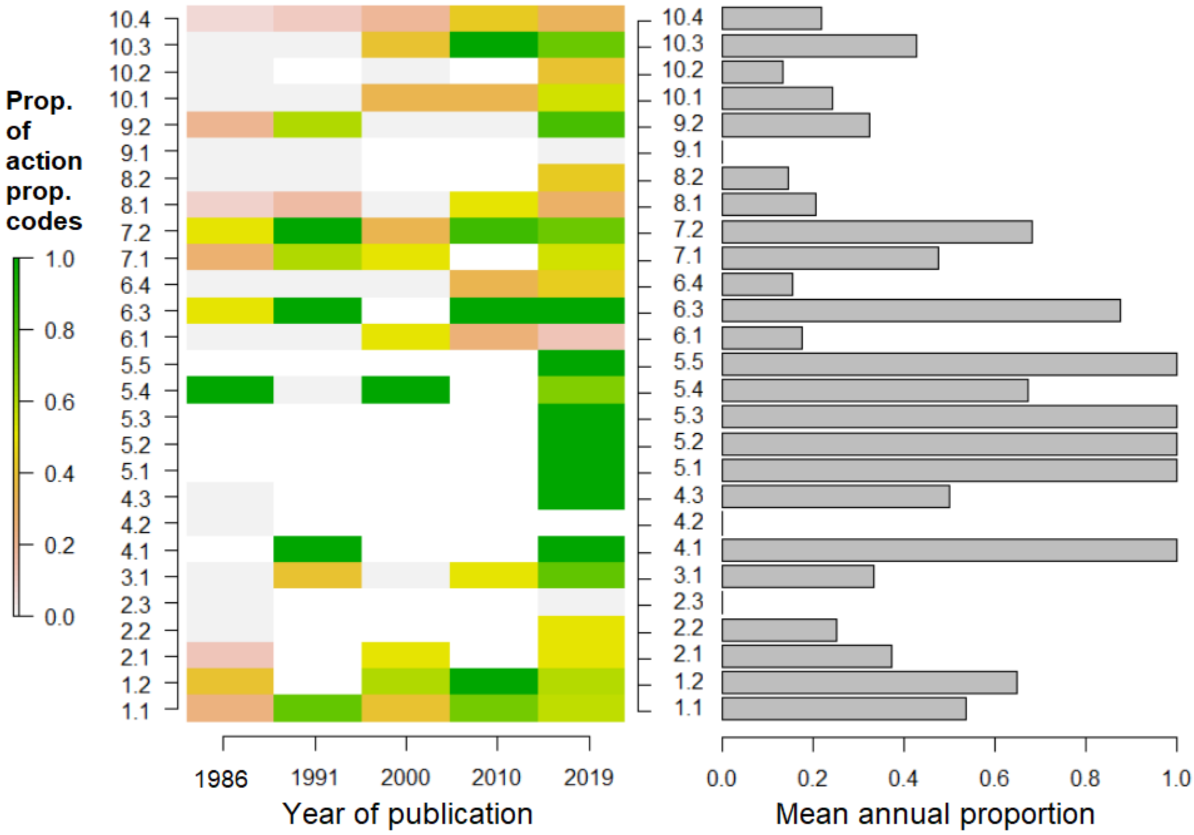

Appendix S12: The proportion of cross-sectoral out of all general action proposal codes of species assessments (colours) over time (x axis, left) and as mean annual proportions per action category (right).

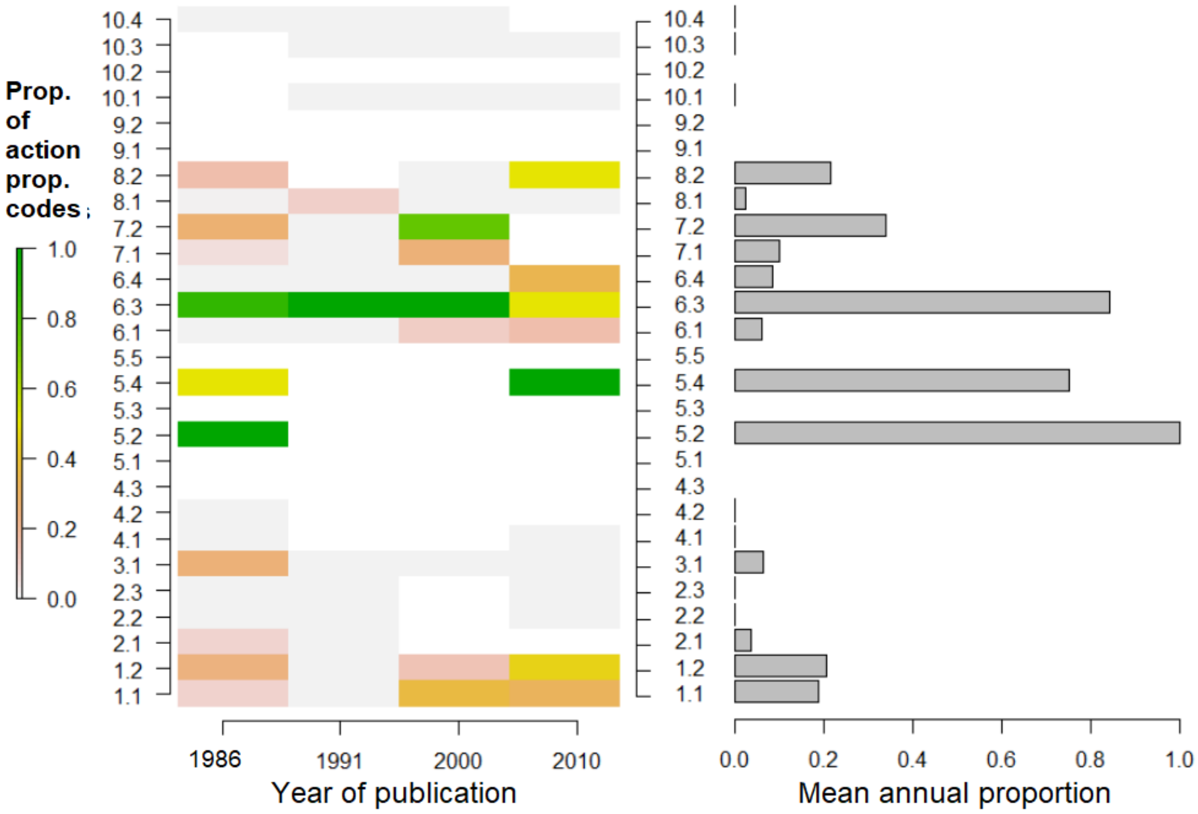

Appendix S13: The proportion of cross-sectoral out of all species-specific action proposal codes of species assessments (colours) over time (x axis, left) and as mean annual proportions per action category (right).

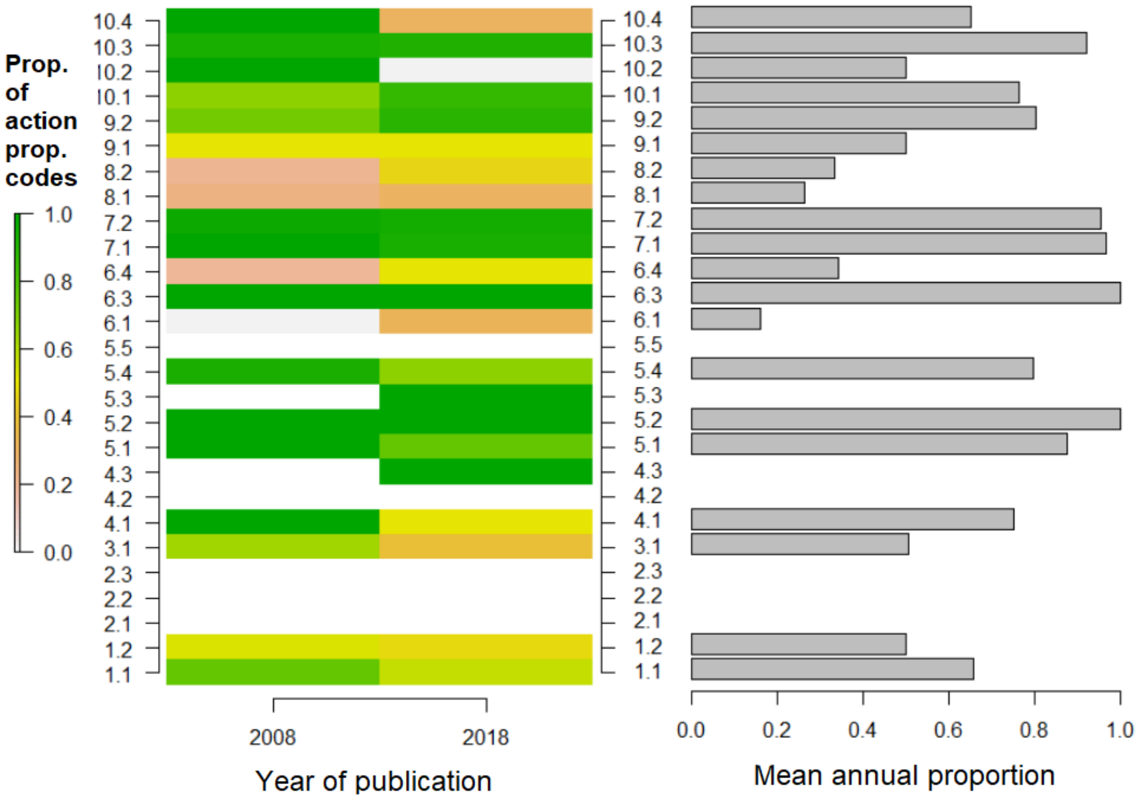

Appendix S14: The proportion of cross-sectoral out of all action proposal codes of ecosystem assessments (colours) over time (x axis, left) and as mean annual proportions per action category (right).
