# Supplementary material for: Changes in the transformative potential of action proposals in Finnish Red Lists from 1986 to 2019
Source: Conserv Biol. 2026 May 6;40(4):e70312. doi: 10.1111/cobi.70312 (PMC13392750; doi:10.1111/cobi.70312)
Supplement: Supplementary file 4 — Supporting information [file COBI-40-e70312-s002.pdf]

# Appendices S15-S17: Numbers and percentages of action categories and leverage points over time in the general action proposals

**Appendix S15. Actions associated with general action proposals.** This table contains the percentage of quotations associated with each action category out of the total number of quotations for each year. The percentages do not sum to 100% because each quotation can be coded under multiple action categories. E.g., 16.7% of general action proposals in 1986 were associated with Site/Area Stewardship. The color codings consist of a gradient from lowest (red) through median (white) to highest (green) values.

| | 1986 | 1991 | 2000 | 2008 | 2010 | 2018 | 2019 |
|---|---|---|---|---|---|---|---|
| 1.1. Site/Area Stewardship | 16.7 | 16 | 19.2 | 10.3 | 31.8 | 9.58 | 31.4 |
| 1.2. Ecosystem & Natural Process (Re)Creation | 4.9 | 0 | 19.2 | 34.5 | 13.6 | 31.7 | 35 |
| 2.1. Species Stewardship | 16.7 | 0 | 7.69 | 0 | 0 | 0 | 3.59 |
| 2.2. Species Re-Introduction & Translocation | 1.96 | 0 | 0 | 0 | 0 | 0 | 2.69 |
| 2.3. Ex-Situ Conservation | 0.98 | 0 | 0 | 0 | 0 | 0 | 0.9 |
| 3.1. Outreach & Communications | 4.9 | 10 | 3.85 | 6.9 | 9.09 | 7.78 | 18.4 |
| 4.1. Detection & Arrest | 0 | 2 | 0 | 2.59 | 0 | 1.2 | 0.45 |
| 4.2. Criminal Prosecution & Conviction | 0.98 | 0 | 0 | 0 | 0 | 0 | 0 |
| 4.3. Non-Criminal Legal Action | 0.98 | 0 | 0 | 0 | 0 | 1.2 | 0.45 |
| 5.1. Linked Enterprises & Alternative Livelihoods | 0 | 0 | 0 | 2.59 | 0 | 2.4 | 2.24 |

| | | | | | | | |
|---|---|---|---|---|---|---|---|
| 5.2. Better Products & Management Practices | 0 | 0 | 0 | 6.03 | 0 | 7.78 | 5.83 |
| 5.3. Market-Based Incentives | 0 | 0 | 0 | 0 | 0 | 1.2 | 2.24 |
| 5.4. Direct Economic Incentives | 1.96 | 2 | 3.85 | 11.2 | 0 | 12.6 | 14.3 |
| 5.5. Non-Monetary Values | 0 | 0 | 0 | 0 | 0 | 0 | 0.45 |
| 6.1. Protected Area Designation &/or Acquisition | 14.7 | 8 | 23.1 | 10.3 | 18.2 | 11.4 | 14.8 |
| 6.3. Land/Water Use Zoning & Designation | 5.88 | 14 | 0 | 12.1 | 27.3 | 15.6 | 6.28 |
| 6.4. Conservation Planning | 7.84 | 8 | 19.2 | 9.48 | 27.3 | 13.2 | 17.5 |
| 7.1. Laws Regulations & Codes | 18.6 | 20 | 7.69 | 9.48 | 0 | 8.38 | 5.83 |
| 7.2. Policies & Guidelines | 3.92 | 14 | 11.5 | 23.3 | 27.3 | 21 | 20.2 |
| 8.1. Basic Research & Status Monitoring | 36.3 | 50 | 42.3 | 30.2 | 45.5 | 26.3 | 28.3 |
| 8.2. Evaluation Effectiveness Measures & Learning | 4.9 | 2 | 0 | 4.31 | 0 | 7.78 | 3.14 |
| 9.1. Formal Education | 0.98 | 2 | 0 | 1.72 | 0 | 1.2 | 0.45 |
| 9.2. Training & Individual Capacity Development | 4.9 | 10 | 3.85 | 6.03 | 4.55 | 5.39 | 12.1 |
| 10.1. Internal Organizational Management & Administration | 8.82 | 18 | 11.5 | 7.76 | 27.3 | 8.38 | 16.6 |
| 10.2. External Organizational Development & Support | 1.96 | 0 | 3.85 | 0.86 | 0 | 1.2 | 2.24 |

| | | | | | | | |
|---|---|---|---|---|---|---|---|
| 10.3. Alliance & Partnership Development | 2.94 | 8 | 19.2 | 12.1 | 13.6 | 6.59 | 17 |
| 10.4. Financing Conservation | 15.7 | 20 | 42.3 | 0.86 | 31.8 | 5.99 | 17.5 |
| Cross-sectoral | 14.7 | 28 | 23.1 | 53.4 | 45.5 | 58.7 | 50.2 |
| Mean number of codings per quotation | 1.9 | 2.3 | 2.6 | 2.6 | 3.2 | 2.7 | 3.3 |

**Appendix S16. Leverage points associated with general action proposals.** This table contains the percentage of quotations associated with each Leverage Point out of the total number of quotations for each year. The percentages do not sum to 100% because each quotation can be associated with multiple Leverage Points. Note that one quotation can have multiple associations to the same LP through several action categories, but these are counted only once. E.g., 9.8% of all general action proposals were associated with LP1 in 1986. The color codings consist of a gradient from lowest (red) through median (white) to highest (green) values

| | 1986 | 1991 | 2000 | 2008 | 2010 | 2018 | 2019 |
|---|---|---|---|---|---|---|---|
| LP1 Transcendence | 9.80 | 16.00 | 7.69 | 11.21 | 13.64 | 12.57 | 22.42 |
| LP2 Paradigms | 30.39 | 40.00 | 30.77 | 30.17 | 22.73 | 26.35 | 39.46 |
| LP3 Goals | 25.49 | 34.00 | 26.92 | 25.86 | 18.18 | 22.75 | 37.22 |
| LP4 System structure | 33.33 | 46.00 | 42.31 | 42.24 | 45.45 | 42.51 | 51.12 |
| LP5 Rules | 40.20 | 44.00 | 46.15 | 50.00 | 54.55 | 55.09 | 57.85 |
| LP6 Information | 75.49 | 92.00 | 80.77 | 71.55 | 90.91 | 73.65 | 81.17 |
| LP7 Driving loops | 48.04 | 60.00 | 69.23 | 63.79 | 72.73 | 67.66 | 77.13 |
| LP8 Control loops | 83.33 | 96.00 | 92.31 | 90.52 | 90.91 | 91.62 | 93.72 |

| | | | | | | | |
|---|---|---|---|---|---|---|---|
| LP9 Delays | 39.22 | 32.00 | 34.62 | 51.72 | 45.45 | 55.09 | 60.09 |
| LP10 Stock structure | 78.43 | 72.00 | 84.62 | 72.41 | 86.36 | 79.64 | 85.65 |
| LP11 Buffers | 78.43 | 72.00 | 84.62 | 72.41 | 86.36 | 79.64 | 85.65 |
| LP12 Parameters | 78.43 | 72.00 | 84.62 | 72.41 | 86.36 | 79.64 | 85.65 |

**Appendix S17. Percentage of general action proposals that were considered cross-sectoral per each action category and assessment.** E.g., 23.53% of general action proposals dealing with Site/area stewardship were cross-sectoral in 1986. The color codings consist of a gradient from lowest (red) through median (white) to highest (green) values.

| | 1986 | 1991 | 2000 | 2008 | 2010 | 2018 | 2019 |
|---|---|---|---|---|---|---|---|
| 1.1. Site/Area Stewardship | 23.53 | 75.00 | 40.00 | 75.00 | 71.43 | 56.25 | 57.14 |
| 1.2. Ecosystem & Natural Process (Re)Creation | 40.00 | | 60.00 | 52.50 | 100.00 | 47.17 | 58.97 |
| 2.1. Species Stewardship | 11.76 | | 50.00 | | | | 50.00 |
| 2.2. Species Re-Introduction & Translocation | 0.00 | | | | | | 50.00 |
| 2.3. Ex-Situ Conservation | 0.00 | | | | | | 0.00 |
| 3.1. Outreach & Communications | 0.00 | 40.00 | 0.00 | 62.50 | 50.00 | 38.46 | 75.61 |
| 4.1. Detection & Arrest | | 100.00 | | 100.00 | | 50.00 | 100.00 |

| | | | | | | | |
|---|---|---|---|---|---|---|---|
| 4.2. Criminal Prosecution & Conviction | 0.00 | | | | | | |
| 4.3. Non-Criminal Legal Action | 0.00 | | | | | 100.00 | 100.00 |
| 5.1. Linked Enterprises & Alternative Livelihoods | | | | 100.00 | | 75.00 | 100.00 |
| 5.2. Better Products & Management Practices | | | | 100.00 | | 100.00 | 100.00 |
| 5.3. Market-Based Incentives | | | | | | 100.00 | 100.00 |
| 5.4. Direct Economic Incentives | 100.00 | 0.00 | 100.00 | 92.31 | | 66.67 | 68.75 |
| 5.5. Non-Monetary Values | | | | | | | 100.00 |
| 6.1. Protected Area Designation &/or Acquisition | 0.00 | 0.00 | 50.00 | 0.00 | 25.00 | 31.58 | 12.12 |
| 6.3. Land/Water Use Zoning & Designation | 50.00 | 100.00 | | 100.00 | 100.00 | 100.00 | 100.00 |
| 6.4. Conservation Planning | 0.00 | 0.00 | 0.00 | 18.18 | 33.33 | 50.00 | 43.59 |
| 7.1. Laws Regulations & Codes | 26.32 | 60.00 | 50.00 | 100.00 | | 92.86 | 53.85 |
| 7.2. Policies & Guidelines | 50.00 | 100.00 | 33.33 | 96.30 | 83.33 | 94.29 | 73.33 |
| 8.1. Basic Research & Status Monitoring | 8.11 | 16.00 | 0.00 | 22.86 | 50.00 | 29.55 | 28.57 |
| 8.2. Evaluation Effectiveness Measures & Learning | 0.00 | 0.00 | | 20.00 | | 46.15 | 42.86 |

| | | | | | | | |
|---|---|---|---|---|---|---|---|
| 9.1. Formal Education | 0.00 | 0.00 | | 50.00 | | 50.00 | 0.00 |
| 9.2. Training & Individual Capacity Development | 20.00 | 60.00 | 0.00 | 71.43 | 0.00 | 88.89 | 81.48 |
| 10.1. Internal Organizational Management & Administration | 0.00 | 0.00 | 33.33 | 66.67 | 33.33 | 85.71 | 54.05 |
| 10.2. External Organizational Development & Support | 0.00 | | 0.00 | 100.00 | | 0.00 | 40.00 |
| 10.3. Alliance & Partnership Development | 0.00 | 0.00 | 40.00 | 92.86 | 100.00 | 90.91 | 73.68 |
| 10.4. Financing Conservation | 6.25 | 10.00 | 18.18 | 100.00 | 42.86 | 30.00 | 30.77 |
