# Supplementary material for: Changes in the transformative potential of action proposals in Finnish Red Lists from 1986 to 2019
Source: Conserv Biol. 2026 May 6;40(4):e70312. doi: 10.1111/cobi.70312 (PMC13392750; doi:10.1111/cobi.70312)
Supplement: Supplementary file 6 — Supporting information [file COBI-40-e70312-s006.pdf]

Appendices S19-S22: Results for the species-specific action proposals

Appendix S19. Results overview

The species-specific proposals had a distinct character with respect to the general proposals, and they originated disproportionately from the first assessment (853 quotations, 54% of total came from the 1986 assessment). Most results were nonetheless similar to the general proposals, but here we focus mainly on those that differed from the general proposals.

The positive trend in complexity was weaker for species-specific action proposals than for the general ones: The mean number of action codings per proposal increased over time from 1.4 to 1.7 (Appendix S20, bottom row). The species-specific proposals addressed the deepest leverage points (1-3) less frequently than general proposals (Appendix S21) largely due to a lower frequency of outreach actions and actions 6-10, which are categorized as "Enabling condition actions" in the original action classification. Contrary to the general proposals, species-specific proposals also did not present an increasing trend in Rules (leverage point LP5) due to the numerous species-specific proposals addressing rules through law-related action proposals especially in 1986 (Appendices S8-S9 and S21). Cross-sectorality was also much lower in species-specific actions with respect to general ones (Appendix S22).

The species-specific action proposals had three main action categories: Basic Research & Status Monitoring (8.1), Protected Area Designation &/or Acquisition (6.1) and Site/Area Stewardship (1.1) that stood out with constantly high frequencies (Appendices S4 and S20). The tendency when focusing on a specific species was to focus on a highly pragmatic and often local level. For example, the 1986 assessment spoke about species under strict protection, and frequently suggested to protect specific habitats or even specific, named locations (73% of *6.1*-codings were in the 1986 assessment), even up to indicating a specific massive oak tree that should be protected in one location. Action proposals regarding *Land or Water Use Zoning and Designation (6.3)* had also a concentration in the 1986 assessment, which stood out with its species-, habitat or location specific zoning proposals.

Appendix S20. Actions associated with species-specific action proposals. This table contains the percentage of quotations associated with each action category out of the total number of quotations for each year. The percentages do not sum to 100% because each quotation can be coded under multiple action categories. E.g., 19.6% of species-specific action proposals in 1986 were associated with Site/Area Stewardship.

	1986	1991	2000	2010
1.1. Site/Area Stewardship	19.6	21.4	22.9	26.0
1.2. Ecosystem & Natural Process (Re)Creation	2.3	10.7	16.7	22.0
2.1. Species Stewardship	13.2	7.1	0.0	0.0
2.2. Species Re-Introduction & Translocation	3.6	3.6	0.0	2.0
2.3. Ex-Situ Conservation	4.0	3.6	0.0	2.0
3.1. Outreach & Communications	0.5	7.1	6.3	4.0
4.1. Detection & Arrest	1.2	0.0	0.0	2.0
4.2. Criminal Prosecution & Conviction	1.2	0.0	0.0	0.0
4.3. Non-Criminal Legal Action	0.0	0.0	0.0	0.0
5.1. Linked Enterprises & Alternative Livelihoods	0.0	0.0	0.0	0.0
5.2. Better Products & Management Practices	0.1	0.0	0.0	0.0
5.3. Market-Based Incentives	0.0	0.0	0.0	0.0
5.4. Direct Economic Incentives	0.3	0.0	0.0	2.0
5.5. Non-Monetary Values	0.0	0.0	0.0	0.0
6.1. Protected Area Designation &/or Acquisition	34.5	25.0	22.9	14.0

6.3. Land/Water Use Zoning & Designation	2.0	3.6	4.2	4.0
6.4. Conservation Planning	5.2	25.0	16.7	6.0
7.1. Laws Regulations & Codes	8.5	7.1	8.3	0.0
7.2. Policies & Guidelines	5.6	3.6	8.3	0.0
8.1. Basic Research & Status Monitoring	32.6	42.9	41.7	52.0
8.2. Evaluation Effectiveness Measures & Learning	0.9	0.0	2.1	4.0
9.1. Formal Education	0.0	0.0	0.0	0.0
9.2. Training & Individual Capacity Development	0.0	0.0	0.0	0.0
10.1. Internal Organizational Management & Administration	0.0	3.6	2.1	8.0
10.2. External Organizational Development & Support	0.0	0.0	0.0	0.0
10.3. Alliance & Partnership Development	0.0	7.1	2.1	8.0
10.4. Financing Conservation	0.1	10.7	2.1	0.0
Cross-sectoral	4.0	14.3	10.4	18.0
Mean number of codings per quotation	1.4	2.0	1.7	1.7

Appendix S21. Leverage points associated with species-specific action proposals. This table contains the percentage of quotations associated with each Leverage Point out of the total number of quotations for each year. The percentages do not sum to 100% because each quotation can be associated with multiple Leverage Points. Note that one quotation can have multiple associations to the same LP through several action categories, but these are counted only once. E.g., 0.53% of all species-specific action proposals were associated with LP1 in 1986.

	1986	1991	2000	2010
LP1 Transcendence	0.53	7.14	6.25	4.00
LP2 Paradigms	9.05	21.43	16.67	12.00
LP3 Goals	9.05	21.43	16.67	12.00
LP4 System structure	12.52	21.43	20.83	12.00
LP5 Rules	48.20	42.86	41.67	20.00
LP6 Information	51.13	82.14	70.83	64.00
LP7 Driving loops	43.68	53.57	52.08	46.00
LP8 Control loops	82.16	82.14	87.50	86.00
LP9 Delays	51.93	50.00	47.92	36.00
LP10 Stock structure	69.11	71.43	68.75	48.00
LP11 Buffers	69.11	71.43	68.75	48.00
LP12 Parameters	69.11	71.43	68.75	48.00

Appendix S22. Percentage of species-specific actions that were considered cross-sectoral per each action category and assessment. E.g., 36.67% of species-specific action proposals dealing with Site/area stewardship were cross-sectoral in 1986.

	1986	1991	2000	2010
1.1. Site/Area Stewardship	36.67	0	80	44.44
1.2. Ecosystem & Natural Process (Re)Creation	13.33	0	20	55.56
2.1. Species Stewardship	23.33	0	0	0
2.2. Species Re-Introduction & Translocation	0	0	0	0
2.3. Ex-Situ Conservation	0	0	0	0
3.1. Outreach & Communications	3.333	0	0	0
4.1. Detection & Arrest	0	0	0	0
4.2. Criminal Prosecution & Conviction	0	0	0	0
4.3. Non-Criminal Legal Action	0	0	0	0
5.1. Linked Enterprises & Alternative Livelihoods	0	0	0	0
5.2. Better Products & Management Practices	3.333	0	0	0
5.3. Market-Based Incentives	0	0	0	0
5.4. Direct Economic Incentives	3.333	0	0	11.11
5.5. Non-Monetary Values	0	0	0	0
6.1. Protected Area Designation &/or Acquisition	3.333	0	20	11.11
6.3. Land/Water Use Zoning & Designation	43.33	25	40	11.11

6.4. Conservation Planning	0	0	0	11.11
7.1. Laws Regulations & Codes	10	0	20	0
7.2. Policies & Guidelines	36.67	0	60	0
8.1. Basic Research & Status Monitoring	3.333	25	0	0
8.2. Evaluation Effectiveness Measures & Learning	3.333	0	0	11.11
9.1. Formal Education	0	0	0	0
9.2. Training & Individual Capacity Development	0	0	0	0
10.1. Internal Organizational Management & Administration	0	0	0	0
10.2. External Organizational Development & Support	0	0	0	0
10.3. Alliance & Partnership Development	0	0	0	0
10.4. Financing Conservation	0	0	0	0
